# Whole genome expression and biochemical correlates of extreme constitutional types defined in Ayurveda

**DOI:** 10.1186/1479-5876-6-48

**Published:** 2008-09-09

**Authors:** Bhavana Prasher, Sapna Negi, Shilpi Aggarwal, Amit K Mandal, Tav P Sethi, Shailaja R Deshmukh, Sudha G Purohit, Shantanu Sengupta, Sangeeta Khanna, Farhan Mohammad, Gaurav Garg, Samir K Brahmachari, Indian Genome Variation Consortium, Mitali Mukerji

**Affiliations:** 1 Genomics and Molecular Medicine, Functional Genomics Unit, Institute of Genomics and Integrative Biology (CSIR), Mall Road, 110007 Delhi, India; 2 Department of Statistics, University of Pune, 411007 Pune, India

## Abstract

**Background:**

Ayurveda is an ancient system of personalized medicine documented and practiced in India since 1500 B.C. According to this system an individual's basic constitution to a large extent determines predisposition and prognosis to diseases as well as therapy and life-style regime. Ayurveda describes seven broad constitution types (*Prakriti*s) each with a varying degree of predisposition to different diseases. Amongst these, three most contrasting types, *Vata*, *Pitta*, *Kapha*, are the most vulnerable to diseases. In the realm of modern predictive medicine, efforts are being directed towards capturing disease phenotypes with greater precision for successful identification of markers for prospective disease conditions. In this study, we explore whether the different constitution types as described in Ayurveda has molecular correlates.

**Methods:**

Normal individuals of the three most contrasting constitutional types were identified following phenotyping criteria described in Ayurveda in Indian population of Indo-European origin. The peripheral blood samples of these individuals were analysed for genome wide expression levels, biochemical and hematological parameters. Gene Ontology (GO) and pathway based analysis was carried out on differentially expressed genes to explore if there were significant enrichments of functional categories among *Prakriti *types.

**Results:**

Individuals from the three most contrasting constitutional types exhibit striking differences with respect to biochemical and hematological parameters and at genome wide expression levels. Biochemical profiles like liver function tests, lipid profiles, and hematological parameters like haemoglobin exhibited differences between *Prakriti *types. Functional categories of genes showing differential expression among *Prakriti *types were significantly enriched in core biological processes like transport, regulation of cyclin dependent protein kinase activity, immune response and regulation of blood coagulation. A significant enrichment of housekeeping, disease related and hub genes were observed in these extreme constitution types.

**Conclusion:**

Ayurveda based method of phenotypic classification of extreme constitutional types allows us to uncover genes that may contribute to system level differences in normal individuals which could lead to differential disease predisposition. This is a first attempt towards unraveling the clinical phenotyping principle of a traditional system of medicine in terms of modern biology. An integration of Ayurveda with genomics holds potential and promise for future predictive medicine.

## Background

Genome wide expression as well as genetic marker studies reveal that most genetic variation is due to inter-individual differences at genetic loci within populations [1-4]. The enormous heterogeneity in expression and sequence variation of genes coupled with genetic network interactions and environmental factors contributes to phenotypic diversity in health and disease. In the Ayurveda system of medicine, predisposition to a disease as well as selection of a preventive and curative regime is primarily based on phenotypic assessment of a person which includes one's body constitution termed "*Prakriti*". *Prakriti *is a consequence of the relative proportion of three entities (*Tri-Doshas*), *Vata *(V), *Pitta *(P) and *Kapha *(K), which are not only genetically determined (*Shukra Shonita*), but also influenced by environment (*Mahabhuta Vikara*), maternal diet and lifestyle (*Matur Ahara Vihara*), and age of the transmitting parents (*Kala-Garbhashaya*) (see Additional File 1). In an individual, the Tri-*Dosha*s work in conjunction and maintain homeostasis throughout the lifetime starting from fertilization. Distinct properties and functions have been ascribed to each *Dosha*. For instance, *Vata *contributes to manifestation of shape, cell division, signaling, movement, excretion of wastes, cognition and also regulates the activities of *Kapha *and *Pitta*. *Kapha *is responsible for anabolism, growth and maintenance of structure, storage and stability. *Pitta *is primarily responsible for metabolism, thermo-regulation, energy homeostasis, pigmentation, vision, and host surveillance. Much as it would sound surprising, but the sanskrit version of the modern terms described above exists in the ancient texts (see Additional File 1). Thus phenotypic diversity, according to Ayurveda, is a consequence of a continuum of relative proportions of *Dosha*s resulting in seven possible constitutional types namely *Vata*, *Pitta*, *Kapha*, *Vata*-*Pitta*, *Pitta*-*Kapha*, *Vata*-*Kapha *and *Vata*-*Pitta*-*Kapha*. Amongst these, the first three are considered as extremes, exhibiting readily recognizable phenotypes, and are more predisposed to specific diseases [5-7].

In an earlier study, correlation of specific HLA-DRB1 polymorphisms with *Prakriti *has been reported [8]. Recently an attempt has also been made to integrate Ayurveda with functional genomics to identify pathways associated with activity of crude and active components of a herb, *Ashwagandha*, which is used for cancer treatment [9]. In the present study, to investigate the Ayurvedic system of phenotypic classification in molecular terms, we examined the possibility of identifying genome wide expression and biochemical differences amongst the *Prakriti *types. We considered gene expression for correlation because it is a better measure of functional variation at the molecular level and can also be mapped more effectively to biological processes and pathways [10,11]. It has also been recently demonstrated that genetic variations underlie variations in gene expression [12-14]. As a first step we analyzed normal healthy individuals belonging to the three most contrasting groups – *Vata*, *Pitta*, and *Kapha*.

## Methods

### Development of questionnaire for Prakriti assessment

A questionnaire for clinical phenotyping was designed on the basis of Ayurvedic literature on phenotypes and methods of *Prakriti *assessment (see Additional File 1). The phenotypic classification, broadly, takes into account criteria for defining anatomical features like body built, body frame, size and symmetry of body parts, physiology, physical endurance and aptitudes. Besides, the questionnaire also captures information pertaining to ethnicity, family history of diseases etc. Each of the questions has multiple options to choose from, and each of the options further refers to it being a property attributed to either V, P or K (see questionnaire as Additional File 2 and Table 1). Individuals who had thin and narrow body frame, weakly developed body build, with irregular appetite, food and bowel habits, difficulty in gaining weight, quick at physical activities, dry skin and hair, and less tolerance for cold temperature were considered as *Vata Prakriti*. Individuals with moderately developed build, high frequency of appetite and thirst, good digestive power, perspiration tendency higher than normal, tolerance for cold weather, moderately mobile with moderate physical strength were identified as *Pitta Prakriti*. Individuals who had broad body frames with well developed body build, tendency to gain weight, low appetite and digestion, preferred to be less mobile, less forgetful and with good healing power and cool temperament, were selected as *Kapha *individuals.

**Table 1:** Distinguishing features of individuals of three contrasting *Prakriti *types *Vata*, *Pitta *and *Kapha *and their disease predisposition as described in the original text.

**S. No**	**Features**	***Vata***	***Pitta***	***Kapha***
1	Body frame	Thin	Medium	Broad
2	Body build and musculature	Weakly developed	Moderate	Well developed
3	Skin	Dry and rough	Soft, thin, with tendency for moles, acne and freckles	Smooth and firm, clear complexion
4	Hair	Dry, thin, coarse and prone to breaks	Thin, soft, oily, early graying	Thick, smooth and firm
5	Weight gain	Recalcitrant	Fluctuating	Tendency to obesity
6	Food and bowel habits	Frequent, variable and irregular	higher capacity for food and water consumption	Low digestive capacity and stable food habits
7	Movements and physical activities	Excessive and quick	Moderate and precise	Less mobile
8	Tolerance for weather	Cold intolerant	Heat intolerant	Endurance for both
9	Disease resistance and healing capacity	Poor	Good	Excellent
10	Metabolism of toxic substances	Moderate	Quick	Poor
11	Communication	Talkative	Sharp, incisive communication with analytical abilities	Less vocal with good communication skills
12	Initiation capabilities	Quick, responsive and enthusiastic	Moderate, upon conviction and understanding	Slow to initiate new things
13	Memory	Quick at grasping and poor retention	Moderate grasping and retention	Slow grasping and Good at retention
14	Ageing	Fast	Moderate	Slow
15	Disease Predisposition/Poor prognosis	Developmental, Neurological, dementia, movement and speech disorders, Arrhythmias	Ulcer, bleeding disorders, Skin diseases	Obesity, diabetes, atherosclerotic conditions

We also developed an automated software for parsing and calculating scores of V, P, K in an individual using a 0/1 against V/P/K for each of the questions depending on a no or yes answer respectively. Cumulative scores of V, P and K is calculated in each individual through the software without the intervention of the Ayurveda expert.

### Clinical phenotyping and identification of volunteers

The identification of individuals of predominant *Prakriti *types were carried out by two Ayurveda physicians (co authors of this paper). In order to avoid any confounding observations due to population stratification the study was conducted on Indo-European speaking large populations predominantly from North India. A preliminary assessment of *Prakriti *was carried out on a total of 850 volunteers, nearly half by each of the two clinicians using subjective assessment and a screening questionnaire. The short-listing of individuals to be recruited for detailed phenotyping was also carried out independently. The short-listed individuals were swapped between the two clinicians and were assessed in detail for their *Prakriti *using the questionnaire (see Additional File 2). These comprised of nearly 120 individuals of predominant *Prakriti *and 200 individuals of heterogeneous *Prakriti*. There was nearly 80% concordance observed in *Prakriti *assessment between two clinicians.

Subsequently 96 unrelated ethnically matched healthy individuals with predominance of either *Vata *(39 individuals), *Pitta *(29) or *Kapha *(28) were identified and included equal numbers of both genders (n = 48 in each case) and belonged to an age group of 18 – 40 years (mean age ~23 ± 4 years).

### Sample collection

Peripheral blood samples of selected individuals were collected using standard procedures following ethical guidelines of Indian Council of Medical Research, India and informed consent of volunteers. Sample collection was carried out following approval of the Institutional Bioethics Committee (IBC). Three hours prior to sample collection all the volunteers were provided a similar diet with no interim intake of food, beverage or smoking. It was ensured that the subject was not ill or under any medication. Blood pressure, pulse, and menstrual cycle, if on, were also recorded.

### Biochemical estimation and analysis

33 biochemical parameters which are used in routine diagnostics were selected. Biochemical estimations were carried out using automated analyzer and standardized kits on all selected volunteers. The tests performed and the standard accepted normal ranges for these parameters are provided in the Additional File 3. In order to determine differences between the groups, the parameters with normal distribution were analysed using one way ANOVA with the help of MATLAB. Kruskal Wallis was used for those with non normal distribution.

### DNA isolation, genotyping and validation of genetic homogeneity

Before undertaking research using these collected samples, the samples were coded in order to maintain their anonymity. Genomic DNA was isolated from peripheral blood leukocytes using the salting-out procedure [15].

In order to validate genetic homogeneity, these samples were genotyped and analyzed along with 24 reference populations derived from different ethnic and linguistic lineages, Indo-European (IE), Austro-Asiatic (AA), Tibeto-Burman (TB) and Dravidian (DR) from various geographical zones using a panel of SNPs which were identified as a part of an ongoing Indian Genome Variation Consortium project[16,17]. Genotyping of SNPs was carried out on the Bead array based Illumina platform. Estimation of DA distance [17,18] between populations and phylogenetic analysis was carried out using the neighbor joining (NJ) method[19] which was done using DISPAN (available from )

### RNA isolation and cDNA microarray experiments

**Figure 1:**
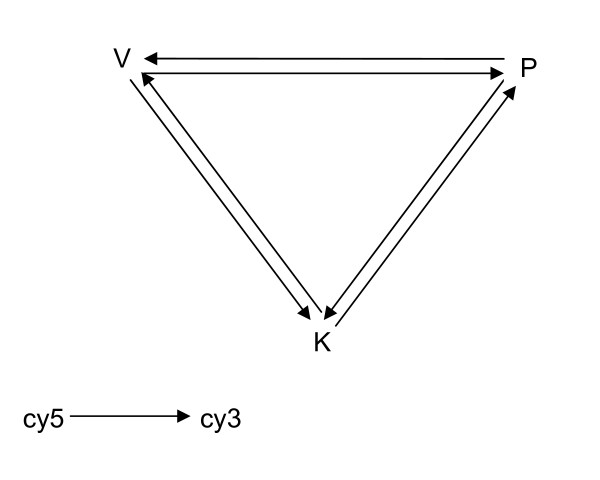
**Loop design of microarray experiment**. V, P, K represent *Vata*, *Pitta*, *Kapha *and arrow head and tail indicate Cy3 and Cy5 respectively. In one set of experiment comprising of three slides each *Prakriti *has been labelled with Cy3 or Cy5 and hybridised to the other two *Prakriti *samples labelled with the opposite dye.

RNA was extracted within 2–3 hrs of collection using the EZ-RNA isolation kit (Biological Industries, Israel) following the manufacturer's protocol. Gene expression profiling was carried out using Human 19Kv8 cDNA microarray (UHN Microarray Databases, CA Ontario). We followed a loop design method for inter group comparisons (illustrated below). In each set of experiments (Figure 1) comprising of 3 slides, pooled samples of each *Prakriti *were labeled with Cy3 and Cy5 and hybridized to the other two [20]. Four sets of experiments for males and females each were carried out involving a total of 72 samples. One set of experiments failed the QC criteria and was not considered for analysis. The results of 4 biological replicates and 4 technical replicates (dye swap) of each *Prakriti *in females, and 3 biological and 3 technical replicates of each *Prakriti *in males are presented. The detailed method for labeling, hybridization and scanning are given below. The data has been submitted to Gene Expression Omnibus (GEO, ID GSE7883) following Minimum Information About Microarray Experiment (MIAME) norms.

Double stranded cDNA was synthesized from 15 μg of total RNA of pooled samples (5 μg each sample), using Microarray cDNA Synthesis Kit (Roche, GmbH). The cDNA was purified using Micorarray Target Purification Kit (Roche, GmbH), according to the manufacturer's protocol. Each pool of *Vata*, *Pitta *and *Kapha *purified cDNA was divided into two halves, and labeled with Cy5 or Cy3 (Amersham Biosciences) using Microarray RNA Target Synthesis Kit T7 (Roche, GmbH) and the labeled products were purified using Microarray Target Purification Kit (Roche, GmbH). Pooled samples were precipitated, washed and air-dried. The dried pellet was dissolved in RNAase free water (Sigma). Hybridization solution was prepared by mixing hybridization buffer (DIG Easy Hyb; Roche, GmbH), 10 mg/ml salmon testis DNA (0.05 mg/ml final concentration, Sigma) and 10 mg/ml yeast tRNA (0.05 mg/ml final concentration, Sigma) and added to the labeled product. This mixture was denatured at 65°C and applied on to 19K cDNA microarray. Hybridization was carried out at 37°C for 16 hrs. Following hybridization, slides were washed thrice (15 minutes each) in 1× SSC (Saline Sodium Citrate) and 0.2% SDS (Sodium Dodecyl Sulphate) at 50°C. This was followed by two 15 minute washes at room temperature in 1× SSC. Finally, slides were washed in 0.1× SSC for 15 minutes and excess fluid was removed from the slide by centrifugation at 600 rpm for 5 minutes. Microarray slides were scanned at 10 μm resolution in GenePix 4000A Microarray Scanner (Molecular Devices). The 16 bit TIFF images were preprocessed and quantified using Gene Pix Pro 6.0 software (Molecular Devices).

### Statistical analysis of microarray

Analysis was carried out using R language [21] taking two sets of experiments at a time (which include four data points each of V, P, K), separately for male and female data. Background subtracted mean values were considered for analysis and only spots having above background values across all arrays were considered. Values were log2 transformed and lowess normalization was applied to remove spatial dye bias. Across-array normalization was carried out using quantile normalization method. One-way analysis of variance was adopted to identify differentially expressed genes in all three categories. F-test was carried out in order to reject the hypothesis of equality of three group means. In order to investigate further the nature of differentially expressed genes; strip charts were drawn and pair wise comparisons were made using t-test. p-values obtained from t-test have been adjusted for multiplicity using Bonferroni method for controlling family wise error rate. Genes at p ≤ 0.05 level of significance were considered to be differentially expressed among all three groups.

In order to investigate whether the expression differences observed between V, P, K were specific or contributed by inter-individual variations we randomly generated three groups from the same experiment sets as used in V, P, K analysis. A similar analysis methodology was applied as described above and genes whose expression differences were significantly different at p ≤ 0.05 were considered. This was repeated twice with different random sets.

In addition we also studied genes which show intra group variations within each constitution types (by estimating variance) as well as those which did not vary across all the constitution types (by estimating S.D). For both the cases quantile normalized expression values were used, treating each pool as a sample. We selected a 5% cut off of upper variance and lower S.D for identifying the genes that show intra group variations or are similar across groups, respectively.

Similarly to investigate whether the biochemical differences obtained among V, P and K are specific to these groups, we randomly labeled the 96 samples into three groups and carried out ANOVA. This process was iterated 1000 times. The mean of the F values obtained from random samples were compared with that obtained with the test set and depicted as Box plot using R language.

### Quantitative PCR analysis

In order to validate the microarray experiment results a small subset of 18 genes along with 18S rRNA (control) were selected for quantitative real time PCR on individual samples of males and females. TAQMAN PCR using custom designed TLDA assay (Applied Biosystems) was carried out on ABI 7900. Each experiment was carried out in triplicates. RNA from all samples were reverse transcribed to cDNA using High Capacity cDNA Archive kit (Applied Biosystems, Foster City, CA), following the manufacturer recommended protocols. The cDNA was amplified using Taqman universal PCR mastermix (Applied Biosystems, Foster City, CA). It was ensured that the amount of cDNA template added to each reaction was restricted to a relatively narrow Ct range as determined by the cDNA quality control measurement of 18S rRNA. Since no single reference/control was used in the study, instead of fold change values, expression differences were estimated through comparison of delta (Δ) Ct values of V, P and K subsets using ANOVA. ΔCt values were calculated for each gene using 18srRNA as internal control. Since the sample sizes were small, both males and females (n = 96) were considered together for the analysis. One way ANOVA was carried out using MATLAB for determining differences between the three and two groups. Low or High mean ΔCt values were inferred as up-regulation or down-regulation respectively.

### Functional annotation of differentially expressed genes

Gene ontology (GO) analysis was carried out using GO tools Box  for classifying the differentially expressed genes into biological processes. Annotation of 19 K array as obtained by source batch search  (June 2007 release) was used for GO analysis. We looked at enriched GO categories both with (p ≤ 0.05), and without Bonferroni correction (p ≤ 0.01) as Bonferroni correction has been proposed to be overly conservative and counterproductive in interpreting microarray results [22]. Pathway analysis was carried out using Pathway Express of ONTO-Tools [23]. We followed the definition of hubs as those which belonged to the top 20% of the interacting proteins [24] as reported in HPRD database . These hubs had more than ten interacting partners. Human housekeeping genes were retrieved from the Eisenberg and Levanon's study [25]. Cluster and Tree View were used for making Heat Maps.

## Results

### Genetic affinity of the study population to Indo-European populations of India

Indian populations exhibit extensive linguistic and ethnic diversity. Therefore, as a first step, the genetic homogeneity of the study population was validated by phylogenetic analysis with known ethnic Indian populations included in the Indian Genome Variation Consortium. Analysis confirmed that the study population was most closely related to Indo-European Large populations [16,17] which corroborated with the information captured in the questionnaire (see Additional File 4).

### Clinical phenotyping

On an average 10% of the individuals were of predominant *Prakriti *types as assessed by the physicians. Cumulative scores of V, P and K calculated for each individual through the software without the intervention of the Ayurveda expert also corroborated with the expert's assessment. The percentage mean score of either V, P or K features in individuals of predominant *Vata*, *Pitta *or *Kapha *constitution types respectively calculated through the software was significantly higher than the mean score of V.P, K obtained from set of heterogeneous *Prakriti *groups who were not classified as predominant types.

### Individuals of different *Prakritis *exhibit differences at biochemical level

**Figure 2:**
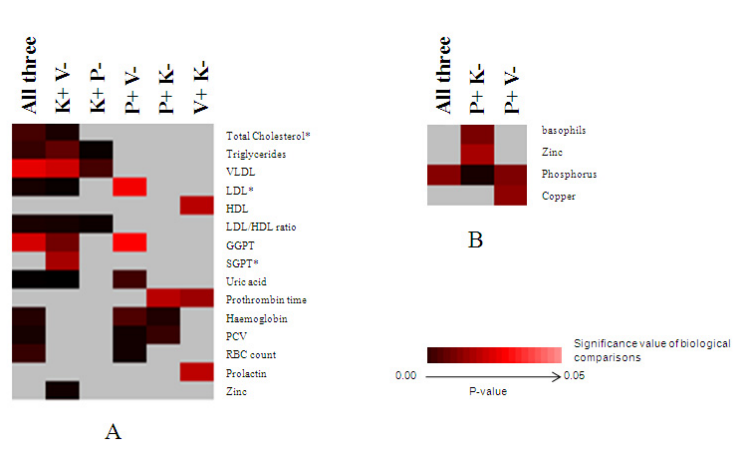
**Differentiating biochemical and hematological profile between and *Prakriti *groups**. Data shown is based on biochemical and hematological profile differences in distinct *Prakriti *groups – "*Vata*" (V), "*Pitta*" (P) and "*Kapha*" (K). Signs "-"and "+" refers to lower and higher values respectively. Heat map (made using Cluster and Tree View) represented in Figures (A) and (B) depicts significant differences in male and female respectively. "*" indicates parameters with non normal distribution. These have been compared using Kruskal-Wallis Test.

Among the 33 Biochemical parameters, 15 parameters in males and 4 in females, revealed significant differences (p ≤ 0.05), albeit, within the normal range, with respect to *Prakriti *(Figure 2A and 2B and see Additional File 5). Notably, the components of the lipid profiles like triglycerides (TG), total cholesterol, VLDL, LDL, LDL/HDL ratio, the common risk factor for cardiovascular diseases was higher in *Kapha *when compared to *Pitta *and *Vata *males. Additionally, *Kapha *also had lower levels of HDL when compared to *Vata*. The levels of serum uric acid, recently considered to be an independent predictor of cardiovascular mortality, were also found to be elevated in *Kapha*. In addition, GGPT, SGPT, and serum Zinc were found to be high in *Kapha*. Serum prolactin and prothrombin time were high in *Vata *in comparison to *Kapha *and/or *Pitta*. On the other hand, hematological parameters like hemoglobin, PCV, and RBC count differed significantly amongst all the three groups where, *Pitta *males showed high values in comparison to *Vata *and/or *Kapha*.

In females only 4 parameters among all those measured differed among the groups with *Pitta *showing the highest levels in all cases. Phosphorus level varied significantly among groups with *Pitta *> *Kapha *> *Vata*. Besides, *Pitta *differed from *Kapha *in basophils and zinc and from *Vata *in copper levels.

Significant differences were not obtained when random sets (1000 iterations) of individuals grouped into three sets were compared. The mean of F statistics from ANOVA of the random sets was insignificant and always lower than the test sets (see Additional File 6).

### Significant differences in genome wide expressions among individuals of distinct *Prakriti *types

Analysis of genome wide expression through cDNA microarrays, using independently pooled samples of *Vata*, *Pitta *and *Kapha *males and females in a set of loop design experiments revealed a number of differentially expressed genes in each category of individuals. Out of the 8416 annotated genes in the 19K array (CA Ontario) 159 in males and 92 in females (excluding un-annotated ESTs) were observed to be differentially expressed (p ≤ 0.05). Only 5 genes among these were common to both the groups. Amongst the differentially expressed genes there was a significant (p < 1.8 × 10–12; n = 53) over-representation of hub and housekeeping genes (p < 10-07; n = 19) (see Additional File 7). 93% of the differentially expressed genes amongst the *Prakriti *groups did not show any overlap with the genes obtained with the random sets. Real time quantitative PCR was carried out on 96 individual samples for validation of microarray data. 18 genes were considered for analysis, eight (ADM, ATP5G2, CH25H, FAS, FTL, HLA-DQB1, KCNJ2, TALDO1) showed similar profiles as observed in microarray (see Additional File 8).

### Systemic differences in *Prakriti *types reflected in GO categories and molecular pathways

Gene Ontology (GO) analysis of differentially expressed genes, revealed contrasting enrichments of core biological processes which remained significant even after Bonferroni correction (p ≤ 0.05, Table 2 and 3) amongst the *Prakriti *groups. Males of the *Vata *group showed a distinct up regulation of genes involved in regulation of cyclin dependent protein kinase activity and regulation of enzyme activity. In *Vata *females, over-expression of genes related to nucleocytoplasmic transport was observed. In *Kapha *males, overall down-regulation of genes of fibrinolysis involved in negative regulation of blood coagulation was observed. *Pitta *males exhibited significant over expression of genes related to immune response (response to biotic stimulus) (Table 2 and 3). However, there were some distinct differences between males and females. Besides, females also demonstrated comparatively lower inter group differences and a considerable higher intra-group variance (data not shown).

**Table 2:** Biochemical parameters, biological processes and pathways that distinguish *Prakriti *types in males

**Distinguishing Parameters (Males)**			***Vata***	***Pitta***	***Kapha***
**Biochemical profiles**	**Lipid profile**	Total Cholesterol*	-	NS	+
		Triglycerides	-	-	+
		VLDL	-	-	+
		LDL*	-	+	+
		HDL	+	NS	-
		LDL/HDL ratio	-	-	+
	**LFT**	GGPT	-	+	+
		SGPT*	-	NS	+
		Prothrombin time	+	+	-
	**Hematological**	Haemoglobin	-	+	-
		PCV	-	+	-
		RBC count	-	+	NS
		Prolactin	+	NS	-
		Uric Acid	-	+	+
		Zinc	-	NS	+
**Biological processes**		immune response	-	+	-
		regulation of enzyme activity	+	-	-
		regulation of transferase activity	+	-	-
		fibrinolysis	+	+	-
**Biological Pathways**	**Environmental Information processing**	Jak-STAT signaling pathway	-	+	-
		Cytokine-cytokine receptor interaction	-	+	-
		Cell adhesion molecules (CAMs)	+	-	-
	**Immune system**	Type I diabetes mellitus	-	+	-
		Natural killer cell mediated cytotoxicity	-	+	-
		Antigen processing and presentation	-	+	-
		B cell and T cell receptor signaling pathway	-	-	+
		Toll-like receptor signaling pathway	-	+	+
		Cell cycle	+	-	-
	**Sensory system**	Taste and olfactory transduction	+	-	+

**Table 3:** Biochemical parameters, biological processes and pathways that distinguish *Prakriti *types in females

**Distinguishing Parameters (Females)**			***Vata***	***Pitta***	***Kapha***
Biochemical		Basophils	NS	+	-
	**Micronutrients**	Zinc	NS	+	-
		Copper	-	+	NS
		Phosphorus	-	+	-
Biological Processes	**Transport**	protein import into nucleus	+	-	-
		NLS-bearing substrate import into nucleus	+	-	-
Biological Pathways	**Immune system**	Toll-like receptor signaling pathway	-	+	-
	**Cellular process**	Apoptosis	-	+	-
		Regulation of actin cytoskeleton	-	-	+
	**Environmental Information processing**	MAPK signaling pathway	-	-	+
		Jak-STAT signaling pathway	-	+	+
		Cytokine-cytokine receptor interaction	-	+	+
	**Sensory system**	Olfactory transduction	+	-	+
	**Diseases**	Epithelial cell signaling in Helicobacter pylori infection	-	+	-
		Neurodegenerative disorders	-	-	+
		Dentatorubropallidoluysian atrophy (DRPLA)	+	+	-

Significantly different biochemical parameters (p ≤ 0.05), enriched biological processes (P ≤ 0.05, Bonferroni corrected) and pathways (p ≤ 0.05) in different *Prakriti *groups in males (A) and females (B). The biological processes were analysed using GO tools and pathway analysis was carried out using Pathway Express of ONTOTOOLs. (+) and (-) indicate high and low levels respectively. NS – non significant.

**Figure 3:**
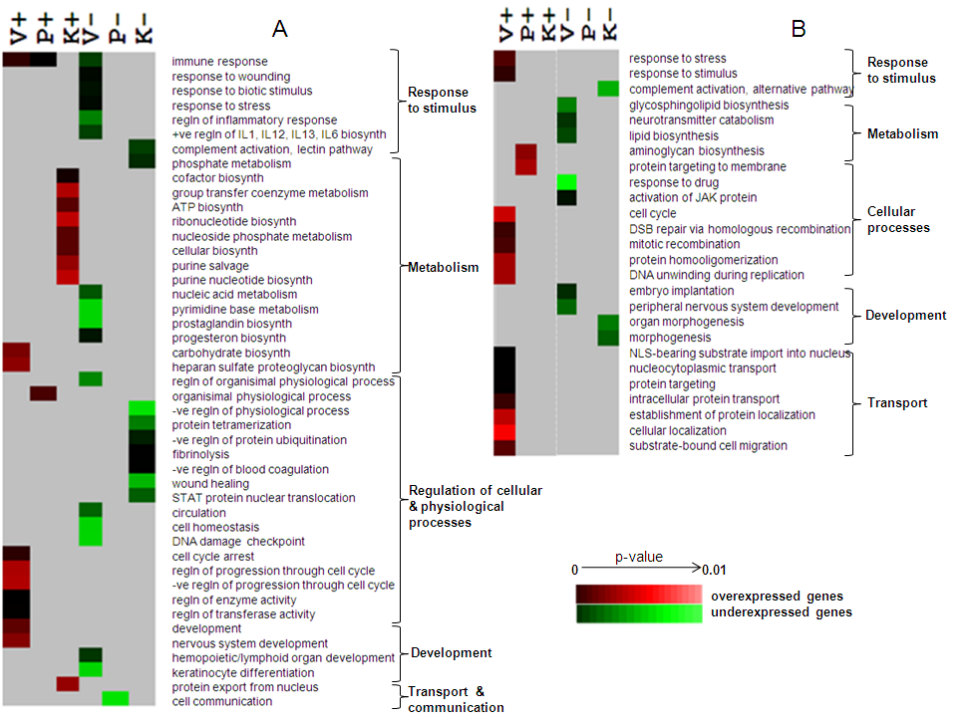
**Differentiating genome wide expression profile between *Prakriti *groups**. Data shown is based on Gene Ontology Biological Process (GOBP) category enrichments in distinct *Prakriti *groups – "*Vata*" (V), "*Pitta*" (P) and "*Kapha*" (K). Each column defines profiles based on their unique expression in one *Prakriti *compared to the other two. For instance "P-" refers to down-regulation in *Pitta *when compared to *Vata *and *Kapha *and vice versa for "P+". Heat map (made using Cluster and Tree View) represented in Figures (A) and (B) depicts significant GOBP enrichments in male and female respectively.

When analysis was done without the overly conservative correction, some similarity in biological processes was revealed in males and females (Figure 3A and 3B). Both males and females of *Vata *group showed enrichment of differentially expressed genes involved in cellular processes like cell cycle, DNA repair and recombination. Additionally males of the *Vata *group showed a distinct down regulation of genes involved in response to biotic stimulus and inflammatory response which was not observed in the corrected GO Biological processes. In *Kapha *males, overall up-regulation of genes involved in cellular biosynthesis including ATP and cofactor biosynthesis and purine salvage pathway were observed. Complement activation was observed to be low in both *Kapha *males and females.

We also compared the biological processes which differentiated the constitution types versus the ones which did not. Strikingly, none of the biological processes obtained after correction show any overlap. However, when we did not perform correction there were a few overlaps in the processes. Importantly, in males, immune response, cell homestasis, co-factor biosynthesis, purine salvage, fibrinolysis, DNA damage check point and protein export from nucleus were exclusive to the differentially expressed set. In females, lipid biosynthesis, neurotransmitter catabolism, NLS-bearing substrate import into nucleus, mitotic recombination was unique to the differentially expressed set. The processes like cell division, differentiation, motility and development as well as processes related to protein localization were observed to be enriched in the dataset of similarly expressed genes (see Additional File 9).

The systemic differences observed between the *Prakriti*s in biological processes were also evident in pathways. For instance over-expression of genes of immune related pathways was observed in *Pitta *and cell cycle related in *Vata *in males (Table 2 and 3). Though immunity was not reflected as enriched in GO process, both B- and T-cell receptor signaling pathways were enriched in over-expressed genes of *Kapha *males. It is interesting to note that functional categories of genes that significantly vary among *Prakriti *groups correlate with few descriptions of their functions in Ayurveda for e.g. *Vata *(cell division and regulation of function of *Kapha *and *Pitta*), *Pitta *(host surveillance) and *Kapha *with anabolism (see Additional File 1)

## Discussion

We observed significant differences in biochemical and genome wide expression levels in individuals from three contrasting constitution types selected on the basis of phenotyping principles of Ayurveda (Table 2 and 3). A number of differences in biochemical parameters also correlate to gene expression differences. Lipid profiles, which are used as markers for cardiovascular diseases differed significantly between groups. Apart from lipid profile, serum uric acid, recently considered to be an independent predictor of cardiovascular mortality [26] was also found to be significantly high in *Kapha *males, compared to other groups. Besides, *Kapha *males also had high levels of LDL, reduced prothrombin time and low expression of genes related to fibrinolysis (KRT1 and F2), features which are reported to increase risk for atherosclerotic conditions [27,28]. Susceptibility of *Kapha *individuals to cardiovascular and atherosclerosis also exists in Ayurveda texts (*Dhamani Pratichaya*, see Additional File 1). Females classified by *Prakriti *did not exhibit differences in lipid profiles, possibly due to age and heterogeneity in hormonal cycles even within similar constitution types. Heterogeneity in females were also reflected in gene expression profiles.

Hematological differences also correlated with gene expression levels. Higher expression of genes which affects hemoglobin levels HBA1, HBB, NOV [29], in *Pitta *compared to *Vata *and *Kapha *correlate with the differences in hemoglobin levels between the *Prakriti*s.

Interestingly, 78 (31% of the entire data set) of the genes that were differentially expressed among *Prakriti *groups were reported to be associated with complex and monogenic diseases in OMIM and genetic association database (GAD). Noteworthy were a number of hub genes like TLR4, FAS, HLA-DQB1, HLA-DQA1, F2, HLA-C, IFNAR1, which link complex diseases related to metabolic, immunological, infectious, cardiovascular, neuro-psychiatric disorders and cancer (see Additional File 10). Additionally genes DPYD [30,31], ABCC1[32], FTL[33] and ICAM3[34] whose expression is shown to be associated with outcome of cancer treatment were also observed in the data set. The differential expression of these disease associated genes might have implication in health and disease.

## Conclusion

It is well acknowledged that subtle variations in large number of genes and their interactions can give rise to system-wide changes which confer differential predisposition to diseases. These variations are common and contribute to 95% of the inter-individual differences observed both at the expression and genetic level in a population. In this exploratory study, we have analysed differences between subsets of individuals from a homogeneous population which are at the phenotypic endpoints of normal health spectrum identified using method of classification described in Ayurveda. The extreme constitution types revealed differences at gene expression level as well as biochemical levels and also included genes with reported disease involvement. Interestingly, it revealed differences in a significant number of hub and housekeeping genes which if perturbed can have system-wide effects. Although, this study has been carried out on a small dataset and requires a more rigorous study of Ayurveda based phenotype to genotype correlations on larger number of individuals and diverse populations for validation, nevertheless, it gives us a lead and confidence to use this method of phenotyping to capture variability among the normal healthy individuals and classify them into more uniform population types.

The study provides a molecular framework for understanding the holistic principles of the Indian Traditional System of Medicine, Ayurveda. Identification of genetic variations that underlie differential expression of genes and biochemical end-points, co-relatable to Prakriti phenotypes will further provide a strong basis for integration of this holistic science with modern genomic approaches for predictive marker discovery and system biology studies.

## Competing interests

The authors declare that they have no competing interests.

## Availability and requirements













## Authors' contributions

SKB conceived the project. MM designed the study. MM and BP planned and implemented the project. BP developed the questionnaire and BP and SA carried out the clinical phenotyping, SK along with BP developed the software for automated generation of scores. SKB and MM provided inputs in questionnaire development. BP, SA, SN and MM recruited volunteers for study and collected the samples. SN carried out the molecular biology experiments including, DNA, RNA isolation, microarray and Real Time (RT) experiments. MF helped with microarray experiments. GG and SS carried out biochemical measurements. Statistical analysis of microarray data was performed by SP and SD, SN did a part of the microarray and RT analysis. TPS and AM assisted in statistical analysis. Global data analysis and interpretation including annotation, GO analysis, biochemical analysis (inputs from SS) and illustrations by BP, SN, SA and MM, BP, SN and MM wrote the paper. SKB provided critical inputs in manuscript. All authors read and approved the final manuscript.

## Supplementary Material

Additional file 1 **Original Sanskrit versions supporting the text**. References from original compendium of Ayurveda, pertaining to the concept of Prakriti, Tridosha and its importance in predictive and curative medicine. Click here for file

Additional file 2 **Questionnaire for *Prakriti *evaluation**. A detailed questionnaire for evaluation of *Prakriti *developed on the basis of original Ayurveda text. Click here for file

Additional file 3 **Normal reference range for biochemical and hematological parameters**. A table of standard accepted normal ranges in males and females for biochemical and hematological parameters. Click here for file

Additional file 4 **Neighbor Joining tree showing relatedness of study population to the Indo European population**. Heterogeneity and inter-relatedness of Indian populations among themselves and with study population (VPK) depicted in neighbour-joining tree illustrating population affinities based on Nei's DA distance. The study population are genetically closer to the Indo-European large populations predominantly from North India. The symbols represents linguistic Lineage (AA, Austro-Asiatic; IE, Indo-European; DR, Dravidian and TB, Tibeto-Burman) followed by geographical location (N, north; NE, north-east; W, west; E, east; S, south and C, central) and Ethnic category (LP, castes/large populations; SP, religious groups/small populations and IP, tribes/isolated population). Click here for file

Additional file 5 **Differences in Biochemical and hematocrit parameters among *Prakriti *groups in males and females**. The data provided shows significant differences (p ≤ 0.05) in biochemical parameters and hematocrit between *Prakriti *groups in males (Table 2) and females (Table 3) as calculated using ANOVA (for values with normal distribution) and Kruskal Wallis (for values with non-normal distribution). Click here for file

Additional file 6 **Validation of biochemical differences amongst three Prakriti groups through analysis of significance performed in 1000 random iterations of same parent dataset in males**. The boxplot of F-values of 1000 randomized comparisons of (A)Total cholesterol, (B) Triglycerides, (C) VLDL, (D) LDL, (E) LDL/HDL, (F) GGPT, (G) Serum Uric acid,(H) Prothrombin Time, (I) Hemoglobin, (J) PCV, (K) RBC, (L) Serum Zinc values. Thick bar indicated median F-value. Red spot is the mean F-value of randomized comparisons and green spot is the comparative mean F-value of V, P, K comparisons. Click here for file

Additional file 7 **List of genes differentially expressed among *Prakriti *types**. The data represents a list of differentially expressed genes in males and females with information on their disease association as obtained from OMIM and GAD and their categorization as HUB and/Or Housekeeping genes. Click here for file

Additional file 8 **Real time validation of differentially expressed genes from microarray experiments**. The data depicts the list of differentially expressed genes validated using quantitative real time PCR with 18S rRNA as internal control. Analysis has been carried out in 96 individual samples and P ≤ 0.05 has been considered significant. Click here for file

Additional file 9 **Significantly enriched biological processes which are similarly expressed across *Prakriti *groups**. The table contains significantly enriched (P ≤ 0.01 after Bonferroni correction) biological processes that do not differ among *Prakriti *groups. Genes which do not differ (as estimated by low S.D) were analysed using GO biological processes. Quantile normalized gene expression values were used to determine SD. Click here for file

Additional file 10 **Disease gene network of differentially expressed genes among *Prakriti *groups**. The figure represents networks of differentially expressed genes and their disease associations obtained from Genetic Association Database (GAD) and OMIM in males (A) and females (B). Networks have been depicted using Cytoscape version 2.4.1 . The nodes colored in yellow represent disease category and those in pink depict gene symbols. Hub genes are represented by pink square nodes. The most connected genes are placed in the centre of the network. Click here for file
