# Supplementary material for: Whole genome expression and biochemical correlates of extreme constitutional types defined in Ayurveda
Source: J Transl Med. 2008 Sep 9;6:48. doi: 10.1186/1479-5876-6-48 (PMC2562368; doi:10.1186/1479-5876-6-48)
Supplement: Additional file 1 — Original Sanskrit versions supporting the text. References from original compendium of Ayurveda, pertaining to the concept of Prakriti, Tridosha and its importance in predictive and curative medicine. [file 1479-5876-6-48-S1.pdf]

Ayurveda describes seven broad constitution (*Prakriti*) types

सप्त प्रकृतयो भवन्ति–दोषैः पृथक्, द्विशः, समस्तैश्च ॥६२॥

su.sa.4/62

Three most contrasting *Prakriti* types are most vulnerable

त्रयस्तु पुरुषा भवन्त्यातुराः, ते त्वनातुरास्तन्त्रान्तरीयाणां भिषजाम् ; तद्यथा—वातलः, पित्तलः, श्लेष्मलश्चेति । तेषामिदं विशेषविज्ञानं—वातलस्य वातनिमित्ताः, पित्तलस्य पित्तनिमित्ताः, श्लेष्मलस्य श्लेष्मनिमित्ता व्याधयः प्रायेण बलवन्तश्च भवन्ति ॥ १५ ॥

c.vi.6/15

***Prakriti* is a consequence of relative proportion of three entities *Vata*, *Pitta* and *Kapha* which is not only genetically determined but also influenced by environment etc**

शुक्रशोणितसंयोगे यो भवेद्दोष उत्कटः ॥
प्रकृतिर्जायते तेन तस्या मे लक्षणं शृणु ॥६३॥

su.sa.4/63

तत्र प्रकृत्यादीन् भावाननुव्याख्यास्यामः । तद्यथा—शुक्रशोणितप्रकृतिं, कालगर्भाशयप्रकृतिं, आतुराहारविहारप्रकृतिं, महाभूतविकारप्रकृतिं च गर्भशरीरमपेक्षते। एतानि हि येन येन दोषेणाधिकेनैकेनानेकेन वा समनुबध्यन्ते, तेन तेन दोषेण गर्भोऽनुबध्यते; ततः सा सा दोषप्रकृतिरुच्यते मनुष्याणां गर्भादिप्रवृत्ता। तस्माच्छ्लेष्मलाः प्रकृत्या केचित्, पित्तलाः केचित्, संसृष्टाः केचित्, समधातवः केचिद्भवन्ति। तेषां हि लक्षणानि व्याख्यास्यामः ॥ ९५ ॥

c.vi.8/95

Vata, ***Pitta*** **and** ***Kapha*** ***doshas*** **work in conjunction and maintain homeostasis throughout life time right after fertilization. These are restrained within normal limits and disease is a perturbation from the threshold.**

नित्याः प्राणभृतां देहे वातपित्तकफास्त्रयः । विकृताः प्रकृतिस्था वा तान् बुभुत्सेत पण्डितः ॥ ४८ ॥

c.su.18/48

सर्वशरीरचरास्तु वातपित्तश्लेष्माणः सर्वस्मिञ्छरीरे कुपिताकुपिताः शुभाशुभानि कुर्वन्ति— प्रकृतिभूताः शुभान्युपचयबलवर्णप्रसादादीनि, अशुभानि पुनर्विकृतिमापन्ना विकारसंज्ञकानि ॥ ९ ॥

c.su.20/9

Distinct functions have been ascribed for *Vata, Pitta* and *Kapha*

उत्साहोच्छ्वासनिःश्वासचेष्टा धातुगतिः समा । समो मोक्षो गतिमतां वायोः कर्माविकारजम् ॥४९॥ → Vata

दर्शनं पक्तिरूष्मा च क्षुत्तृष्णा देहमार्दवम् । प्रभा प्रसादो मेधा च पित्तकर्माविकारजम् ॥ ५० ॥ → Pitta

स्नेहो बन्धः स्थिरत्वं च गौरवं वृषता बलम् । क्षमा धृतिरलोभश्च कफकर्माविकारजम् ॥ ५१ ॥ → Kapha

c.su.18/42

Vata

यानि तु खलु वायोः कुपिताकुपितस्य शरीराशरीरचरस्य शरीरेषु चरतः कर्माणि बहिःशरीरेभ्यो वा भवन्ति, तेषामवयवान् प्रत्यक्षानुमानोपदेशैः साधयित्वा नमस्कृत्य वायवे यथाशक्ति प्रवक्ष्यामः—वायुस्तन्त्रयन्त्रधरः, प्राणोदानसमानव्यानापानात्मा, प्रवर्तकश्चेष्टानामुच्चावचानां, नियन्ता प्रणेता च मनसः, सर्वेन्द्रियाणामुद्योजकः, सर्वेन्द्रियार्थानामभिवोढा, सर्वशरीरधातुव्यूहकरः, सन्धानकरः शरीरस्य, प्रवर्तको वाचः, प्रकृतिः स्पर्शशब्दयोः, श्रोत्रस्पर्शनयोर्मूलं, हर्षोत्साहयोर्योनिः, समीरणोऽग्नेः, दोषसंशोषणः, क्षेप्ता बहिर्मलानां, स्थूलाणुस्रोतसां भेत्ता, कर्ता गर्भाकृतीनाम्, आयुषोऽनुवृत्तिप्रत्ययभूतो भवत्यकुपितः । कुपितस्तु खलु शरीरे शरीरं नानाविधैर्विकारैरुपतपति बलवर्णसुखायुषामुपघाताय, मनो व्याहर्षयति, सर्वेन्द्रियाण्युपहन्ति, विनिहन्ति गर्भान् विकृतिमापादयत्यतिकालं वा धारयति, भयशोकमोहदैन्यातिप्रलापाञ्जनयति, प्राणांश्चोपरुणद्धि ।

Pitta

मरीचिरुवाच—अग्निरेव शरीरे पित्तान्तर्गतः कुपिताकुपितः शुभाशुभानि करोति; तद्यथा—पक्तिमपक्तिं दर्शनमदर्शनं मात्रामात्रत्वमूष्मणः प्रकृतिविकृतिवर्णौ शौर्यं भयं क्रोधं हर्षं मोहं प्रसादमित्येवमादीनि चापराणि द्वन्द्वानीति ॥ ११ ॥

Kapha

तच्छ्रुत्वा मरीचिवचः काप्य उवाच—सोम एव शरीरे श्लेष्मान्तर्गतः कुपिताकुपितः शुभाशुभानि करोति; तद्यथा—दार्ढ्यं शैथिल्यमुपचयं कार्श्यमुत्साहमालस्यं वृषतां क्लीबतां ज्ञानमज्ञानं बुद्धिं मोहमेवमादीनि चापराणि द्वन्द्वानीति ॥ १२ ॥

c.su.12/8/11/12

Basic Constitution or *Prakriti* determines the therapy, diet and lifestyle regime

आतुरस्तु खलु कार्यदेशः। तस्य परीक्षा आयुषः प्रमाणज्ञानहेतोर्वा स्याद्, बलदोषप्रमाणज्ञानहेतोर्वा। तत्र तावदियं बलदोषप्रमाणज्ञानहेतोः; दोषप्रमाणानुरूपो हि भेषजप्रमाणविकल्पो बलप्रमाणविशेषापेक्षो भवति। सहसा ह्यतिबलमौषधमपरीक्षकप्रयुक्तमल्पबलमातुरमतिपातयेत्; न ह्यतिबलान्याग्नेयवायवीयान्यौषधान्यग्निक्षारशस्त्रकर्माणि वा शक्यन्तेऽल्पबलैः सोढुम्; असह्यातितीक्ष्णवेगत्वाद्धि तानि सद्यःप्राणहराणि स्युः। एतच्चैव कारणमपेक्षमाणा हीनबलमातुरमविषादकरैर्मृदुसुकुमारप्रायैरुत्तरोत्तरगुरुभिरविभ्रमैरनात्ययिकैश्चोपचरन्त्यौषधैः; विशेषतश्च नारीः, ता ह्यनवस्थितमृदुविवृतविक्लवहृदयाः प्रायः सुकुमार्योऽबलाः परसंस्तभ्याश्च। तथा बलवति बलवद्व्याधिपरिगते स्वल्पबलमौषधमपरीक्षकप्रयुक्तमसाधकमेव भवति। तस्मादातुरं परीक्षेत प्रकृतितश्च, विकृतितश्च, सारतश्च, संहननतश्च, प्रमाणतश्च, सात्म्यतश्च, सत्त्वतश्च, आहारशक्तितश्च, व्यायामशक्तितश्च, वयस्तश्चेति, बलप्रमाणविशेषग्रहणहेतोः ॥ ९४ ॥

therapy

c.vi.8/94

समपित्तानिलकफाः केचिद्गर्भादि मानवाः। दृश्यन्ते वातलाः केचित्पित्तलाः श्लेष्मलास्तथा ॥ ३९ ॥
तेषामनातुराः पूर्वे वातलाद्याः सदातुराः। दोषानुशयिता ह्येषां देहप्रकृतिरुच्यते ॥ ४० ॥
विपरीतगुणस्तेषां स्वस्थवृत्तेर्विधिर्हितः। समसर्वरसं सात्म्यं समधातोः प्रशस्यते ॥ ४१ ॥

Diet and lifestyle

c.vi.7/39 - 41

Phenotypes of three most contrasting constitution types (Table 1)

Vata	वातस्तु रूक्षलघुचलबहुशीघ्रशीतपरुषविशदः । तस्य रौक्ष्याद्वातला रूक्षापचिताल्पशरीराः प्रततरूक्षक्षामसन्नसक्तजर्जरस्वरा जागरूकाश्च भवन्ति, लघुत्वाल्लघुचपलगतिचेष्टाहारव्याहाराः, चलत्वादनवस्थितसन्ध्यक्षिभ्रूहन्वोष्ठजिह्वाशिरःस्कन्धपाणिपादाः, बहुत्वाद्बहुप्रलापकण्डरासिराप्रतानाः, शीघ्रत्वाच्छीघ्रसमारम्भक्षोभविकाराः शीघ्रत्रासरागविरागाः श्रुतग्राहिणोऽल्पस्मृतयश्च, शैत्याच्छीतासहिष्णवः प्रततशीतकोद्वेपकस्तम्भाः, पारुष्यात् परुषकेशश्मश्रुरोमनखदशनवदनपाणिपादाः, वैशद्यात् स्फुटिताङ्गावयवाः सततसन्धिशब्दगामिनश्च भवन्तिः त एवं गुणयोगाद्वातलाः प्रायेणाल्पबलाश्चाल्पायुषश्चाल्पापत्याश्चाल्पसाधनाश्चाल्पधनाश्च भवन्ति ॥ ९८ ॥
Pitta	पित्तमुष्णं तीक्ष्णं द्रवं विस्रमम्लं कटुकं च । तस्यौष्ण्यात् पित्तला भवन्त्युष्णासहा, उष्णमुखाः, सुकुमारावदातगात्राः, प्रभूतपिप्लुव्यङ्गतिलपिडकाः, क्षुत्पिपासावन्तः, क्षिप्रवलीपलितखालित्यदोषाः, प्रायो मृद्वल्पकपिलश्मश्रुलोमकेशाश्च; तैक्ष्ण्यात्तीक्ष्णपराक्रमाः, तीक्ष्णाग्नयः, प्रभूताशनपानाः, क्लेशासहिष्णवो, दन्दशूकाः, द्रवत्वाच्छिथिलमृदुसन्धिमांसाः, प्रभूतसृष्टस्वेदमूत्रपुरीषाश्च; विस्रत्वात् प्रभूतपूतिकक्षास्यशिरःशरीरगन्धाः; कट्वम्लत्वादल्पशुक्रव्यवायापत्याः; त एवंगुणयोगात् पित्तला मध्यबला मध्यायुषो मध्यज्ञानविज्ञानवित्तोपकरणवन्तश्च भवन्ति ॥ ९७ ॥
Kapha	श्लेष्मा हि स्निग्धश्लक्ष्णमृदुमधुरसारसान्द्रमन्दस्तिमितगुरुशीतविज्जलाच्छः । तस्य स्नेहाच्छ्लेष्मलाः स्निग्धाङ्गाः, श्लक्ष्णत्वाच्छ्लक्ष्णाङ्गाः, मृदुत्वाद्दृष्टिसुखसुकुमारावदातगात्राः; माधुर्यात् प्रभूतशुक्रव्यवायापत्याः, सारत्वात् सारसंहतस्थिरशरीराः, सान्द्रत्वादुपचितपरिपूर्णसर्वाङ्गाः, मन्दत्वान्मन्दचेष्टाहारव्याहाराः, स्तैमित्यादशीघ्रारम्भक्षोभविकाराः, गुरुत्वात् साराधिष्ठितावस्थितगतयः, शैत्यादल्पक्षुत्तृष्णासंतापस्वेददोषाः, विज्जलत्वात् सुश्लिष्टसारसन्धिबन्धनाः, तथाऽच्छत्वात् प्रसन्नदर्शनाननाः प्रसन्नस्निग्धवर्णस्वराश्च भवन्ति । त एवंगुणयोगाच्छ्लेष्मला बलवन्तो वसुमन्तो विद्यावन्त ओजस्विनः शान्ता आयुष्मन्तश्च भवन्ति ॥ ९६ ॥

c.vi.8/96-98

Vata regulates cell division and morphogenesis

शरीरावयवास्तु परमाणुभेदेनापरिसंख्येया भवन्ति, अतिबहुत्वादतिसौक्ष्म्यादतीन्द्रियत्वाच्च। तेषां संयोगविभागे परमाणूनां कारणं वायुः कर्मस्वभावश्च ॥ १७ ॥

c.sa.7/17

Eighty diseases which uniquely involve *Vata* are described in the text below. *Vata Prakriti* individuals are most prone to these diseases. A few examples mentioned in the Table1 are underlined.

तत्रादौ वातविकाराननुव्याख्यास्यामः। तद्यथा—नखभेदश्च, विपादिका च, पादशूलं च, पादभ्रंशश्च, पादसुप्तता च, वातखुड्डता च, गुल्फग्रहश्च, पिण्डिकोद्वेष्टनं च, गृध्रसी च, जानुभेदश्च, जानुविश्लेषश्च, ऊरुस्तम्भश्च, ऊरुसादश्च, पाङ्गुल्यं च, गुदभ्रंशश्च, गुदार्तिश्च, वृषणाक्षेपश्च, शेफस्तम्भश्च, वङ्क्षणानाहश्च, श्रोणिभेदश्च, विड्भेदश्च, उदावर्तश्च, खञ्जत्वं च, कुब्जत्वं च, वामनत्वं च, त्रिकग्रहश्च, पृष्ठग्रहश्च, पार्श्वावमर्दश्च, उदरावेष्टश्च, हृन्मोहश्च, हृद्द्रवश्च, वक्षउद्धर्षश्च, वक्षउपरोधश्च, वक्षस्तोदश्च, बाहुशोषश्च, ग्रीवास्तम्भश्च, मन्यास्तम्भश्च, कण्ठोद्ध्वंसश्च, हनुभेदश्च, ओष्ठभेदश्च, अक्षिभेदश्च, दन्तभेदश्च, दन्तशैथिल्यं च, मूकत्वं च, वाक्सङ्गश्च, कषायास्यता च, मुखशोषश्च, अरसज्ञता च, घ्राणनाशश्च, कर्णशूलं च, अशब्दश्रवणं च, उच्चैःश्रुतिश्च, बाधिर्यं च, वर्त्मस्तम्भश्च, वर्त्मसङ्कोचश्च, तिमिरं च, अक्षिशूलं च, अक्षिव्युदासश्च, भ्रूव्युदासश्च, शङ्खभेदश्च, ललाटभेदश्च, शिरोरुक् च, केशभूमिस्फुटनं च, अर्दितं च, एकाङ्गरोगश्च, सर्वाङ्गरोगश्च, पक्षवधश्च, आक्षेपकश्च, दण्डकश्च, तमश्च, भ्रमश्च, वेपथुश्च, जृम्भा च, हिक्का च, विषादश्च, अतिप्रलापश्च, रौक्ष्यं च, पारुष्यं च, श्यावारुणावभासता च, अस्वप्नश्च, अनवस्थितचित्तत्वं च; इत्यशीतिर्वातविकारा वातविकाराणामपरिसंख्येयानामाविष्कृततमा व्याख्याताः ॥ ११ ॥

Arrhythmia

Speech disorders

Developmental anomalies

Neurological

Psychiatric

c.su.20/11

Forty diseases which uniquely involve *Pitta* are described in the text below. *Pitta Prakriti* individuals are most prone to these diseases. A few examples mentioned in the Table1 are underlined.

पित्तविकारांश्चत्वारिंशतमत ऊर्ध्वमनुव्याख्यास्यामः—ओषश्च, प्लोषश्च, दाहश्च, दवथुश्च, धूमकश्च,
अम्लकश्च, विदाहश्च, अन्तर्दाहश्च, अंसदाहश्च, ऊष्माधिक्यं च, अतिस्वेदश्च (अङ्गस्वेदश्च), अङ्गगन्धश्च,
अङ्गावदरणं च, शोणितक्लेदश्च, मांसक्लेदश्च, त्वग्दाहश्च, (मांसदाहश्च), त्वगवदरणं च, Skin diseases
चर्मदलनं च, रक्तकोठश्च, रक्तविस्फोटश्च रक्तपित्तं च रक्तमण्डलानि च, हरितत्वं च, हारिद्रत्वं च, bleeding disorders
नीलिका च, कक्षा(क्ष्या) च, कामला च, तिक्तास्यता च, लोहितगन्धास्यता च, पूतिमुखता च, तृष्णाधिक्यं
च, अतृप्तिश्च, आस्यविपाकश्च, गलपाकश्च, अक्षिपाकश्च, गुदपाकश्च, मेढ्रपाकश्च, जीवादानं च, तमःप्रवेशश्च ulcers
हरितहारिद्रनेत्रमूत्रवर्चस्त्वं च; इति चत्वारिंशत्पित्तविकाराः पित्तविकाराणामपरिसंख्येयानामाविष्कृत-
तमा व्याख्याताः ॥ १४ ॥

c.su.20/14

Twenty diseases which uniquely involve *Kapha* are described in the text below. *Kapha Prakriti* individuals are most prone to these diseases. A few examples mentioned in the Table1 are underlined.

श्लेष्मविकारांश्च विंशतिमत ऊर्ध्वं व्याख्यास्यामः; तद्यथा—तृप्तिश्च, तन्द्रा च, निद्राधिक्यं च, स्तैमित्यं च, गुरुगात्रता च, आलस्यं च, मुखमाधुर्यं च, मुखस्रावश्च, श्लेष्मोद्गिरणं च, मलस्याधिक्यं च, बलासकश्च, अपक्तिश्च, हृदयोपलेपश्च, कण्ठोपलेपश्च, धमनीप्रति(वि)चयश्च, गलगण्डश्च, अतिस्थौल्यं च, शीताग्निता च, उदर्दश्च, श्वेतावभासता च, श्वेतमूत्रनेत्रवर्चस्त्वं च; इति विंशतिः श्लेष्मविकाराः श्लेष्मविकाराणामपरिसंख्येयानामाविष्कृततमा व्याख्याता भवन्ति ॥ १७ ॥

c.su.20/17

Atherosclerotic conditions, obesity
