# Supplementary material for: Whole genome expression and biochemical correlates of extreme constitutional types defined in Ayurveda
Source: J Transl Med. 2008 Sep 9;6:48. doi: 10.1186/1479-5876-6-48 (PMC2562368; doi:10.1186/1479-5876-6-48)
Supplement: Additional file 2 — Questionnaire for Prakriti evaluation. A detailed questionnaire for evaluation of Prakriti developed on the basis of original Ayurveda text. [file 1479-5876-6-48-S2.pdf]

Questionnaire for *Prakriti* evaluation

Personal information

Language and Ethnicity

Gender

Age

Height

Weight

Family history of Disease

.Vital Data

(a) Pulse Rate/min:

(b) Blood Pressure (mmhg)

Systolic **Diastolic**

(c) Wrist Circumference (inch)

(d) Body frame by Wrist finger ratio

(d) Waist Circumference (inch)

(e) Hip Circumference (inch)

(f) Forearm measurement (inch)

(g) Waist-Hip ratio

(h) B.M.I.

A. Anatomical Features

A-1

	Symmetry		Shape		Length/Height			Breadth		
	Proportionate	Disproportionate	Regular	Irregular	Too Short / Too Long	Medium	Long	Thin / Narrow	Medium	Broad
Body Build	◯	◯								
Body Frame	◯	◯	◯	◯	◯	◯	◯	◯	◯	◯
Head	◯	◯	◯	◯						
Forehead	◯	◯			◯	◯	◯	◯	◯	◯
Face	◯	◯	◯	◯						
Eyebrows			◯	◯						
Eyes	◯	◯	◯	◯						
Lips			◯	◯						
Jaws			◯	◯						
Shoulder			◯	◯				◯	◯	◯
Chest	◯	◯	◯	◯				◯	◯	◯
Hands	◯	◯			◯	◯	◯			
Palms	◯	◯								
Nails					◯	◯	◯			
Legs	◯	◯			◯	◯	◯			
Soles	◯	◯								
Joints	◯	◯								

A-3 Skin

Nature		Texture		Appearance		Colour	
Dry	◯	Smooth	◯	Cracked	◯	Fair	◯
Oily	◯	Firm	◯	Wrinkled	◯	Dark	◯
Normal	◯	Lustrous	◯	Moles	◯	Reddish	◯
Seasonal/Variable	◯	Soft	◯	Marks	◯	Pale Yellow	◯
Thick	◯	Loose	◯	Pimples	◯	Pink	◯
Thin	◯	Rough	◯	Freckles	◯	Wheatish	◯
Hard	◯	Coarse	◯	Clear	◯	Golden	◯
				Prominent Veins	◯	Fresh Colour	◯
						Dusky	◯

A-4 Scalp hair

Texture		Nature		Growth /Bulk	
Thick	◯	Graying	◯	Dense	◯
Thin	◯	Falling	◯	Scanty	◯
Coarse	◯	Breaking	◯	Moderate	◯
Hard	◯	None	◯	Bald	◯
Smooth	◯	Dry	◯		
Soft	◯	Oily	◯		
Straight	◯	Normal	◯		
Wavy	◯	Seasonal/Variable	◯		
Fizzy/Curly	◯				

A-5

Colour	Scalp hair	Body hair
Black	◯	◯
Dark Brown	◯	◯
Light Brown	◯	◯
Dusky	◯	◯
Blonde	◯	
Copper	◯	◯

A-2 Size / Bulk / Musculature

	Small / Weakly developed/ thin	Medium / Moderately developed	Large / Big / Well developed
Body Build	◯	◯	◯
Joints	◯	◯	◯
Face	◯	◯	◯
Eyes	◯	◯	◯
Eyebrows	◯	◯	◯
Eyelashes	◯	◯	◯
Lips	◯	◯	◯

A-6 Eye

Colour		Appearance	
Black	◯	Dry/Dull	◯
Dark Brown	◯	Dim in luster	◯
Light Brown	◯	Shiny	◯
Greyish	◯	Milky white sclera	◯
Blue	◯	Reddish tinge to sclera	◯
Green	◯	Muddy sclera	◯

A-7 Teeth

Size		Appearance		Shape		Color	
Too small	◯	Brittle/ Cracked	◯	Regular	◯	Milky white	◯
Too large	◯	Loose	◯	Irregular	◯	Yellowish	◯
Medium	◯	Lustrous	◯	Even	◯	Dull / blackish	◯
Large	◯			Uneven	◯		

A-8 Complexion

	Colour				Nature							
	Dark	Reddish	Pale Yellow	Pink	Smooth	Firm	Soft	Rough	Brittle / Cracked / Split	Wrinkled	Flat	Convex
Palms	◯	◯	◯	◯	◯	◯	◯	◯	◯	◯		
Soles	◯	◯	◯	◯	◯	◯	◯	◯	◯	◯		
Lips	◯	◯	◯	◯	◯	◯	◯	◯	◯	◯		
Nails	◯	◯	◯	◯	◯	◯	◯	◯	◯		◯	◯
Palate	◯	◯	◯	◯								

B. Physiological Functions

B-1 Metabolism

	Frequency						Amount			
	Regular	Irregular	Frequent	Infrequent	Medium	Variable	Low	High	Medium	Variable
Appetite	◯	◯	◯	◯	◯	◯	◯	◯	◯	◯
Thirst	◯	◯	◯	◯	◯	◯	◯	◯	◯	◯
Bladder	◯	◯	◯	◯	◯	◯	◯	◯	◯	◯

B-2

	Amount			
	Low	High	Medium	Variable
Digestive Power	◯	◯	◯	◯
Perspiration	◯	◯	◯	◯
Body Temperature	◯	◯	◯	◯
Sleep	◯	◯	◯	◯
Dreams	◯	◯	◯	◯

B-3 Bowel habits

Frequency		Tendency towards	
Regular	◯	Constipation	◯
Irregular	◯	Loose motion	◯
Variable	◯	None	◯

Stool consistency

Loose / Soft / SemiSolid	◯	Medium	◯	Hard	◯

B-4 Body odour

Strong	◯
Mild	◯
Very less	◯

B-5 Quality of sleep

Deep	◯
Sound	◯
Shallow	◯

B-6 Body weight changes

Gain and lose easily	◯
Difficulty in gaining	◯
Gain easily and lose with difficulty	◯
Stable	◯

B-7 Food

	Sweet	Sour	Salty	Bitter	Pungent	Astringent	Cold	Warm	Dry	Oily
Like	◯	◯	◯	◯	◯	◯	◯	◯	◯	◯
Does Not Like	◯	◯	◯	◯	◯	◯	◯	◯	◯	◯
Suit	◯	◯	◯	◯	◯	◯	◯	◯	◯	◯
Does Not Suit	◯	◯	◯	◯	◯	◯	◯	◯	◯	◯

B-8

Weather	Prefer	Have health problems
Cold	◯	◯
Warm	◯	◯
Dry	◯	◯
Moist	◯	◯
Moderate	◯	◯
Stable	◯	◯

B-9

Season	Prefer	Have health problems
Summer	◯	◯
Early winter	◯	◯
Late winter	◯	◯
Autumn	◯	◯
Spring	◯	◯
Rainy season	◯	◯
Season transition	◯	◯
None	◯	◯

C. Physical Activities

C-1 Walking

Speed		Steps		Amount		Style	
Quick / Fast / Brisk	◯	Small	◯	Less	◯	Firm / Steady	◯
Medium	◯	Medium	◯	High / Excessive	◯	Unsteady	◯
Slow	◯	Large	◯	Moderate	◯	Sharp / accurate	◯
Variable	◯						

C-2 Working

Speed		Quality		Style / Accuracy	
Quick/Fast/Brisk	◯	Well thought of	◯	Firm / Steady	◯
Medium	◯	Wavering / Easily deviated	◯	Unsteady	◯
Slow	◯	Sharp/Accurate/Spontaneous	◯	Sharp / Accurate	◯
Variable	◯				

C-3 Voluntary / Involuntary movements of body parts

	Eyes	Eyebrows	Jaw	Lips	Tongue	Head	Shoulder	Hands	Legs
Less	◯	◯	◯	◯	◯	◯	◯	◯	◯
Moderate	◯	◯	◯	◯	◯	◯	◯	◯	◯
High / Excessive	◯	◯	◯	◯	◯	◯	◯	◯	◯

C-4

Quality of voice				Content of speech			
Low	◯	Good tone	◯	Consistent	◯	Convincing	◯
Feeble	◯	Sharp	◯	Inconsistent	◯	Argumentative	◯
Weak	◯	Clear	◯	Moderate	◯	Sweet and pleasing to ears	◯
Broken	◯	High pitched	◯	Well guarded / Well thought of	◯	Avoid confrontations	◯
Rough	◯	Loud	◯	Wavering / Easily Deviated	◯	Deviated from main topic	◯
Deep	◯	Soft, pleasing	◯	Sharp / Accurate / Spontaneous	◯	Irrelevant in between	◯

C-5 Speaking

Speed		Amount	
Quick / Fast / Brisk	◯	Less	◯
Medium	◯	Moderate	◯
Slow	◯	Excessive	◯
Variable	◯		

D. Strength at Various Levels

D-1

Physical		Mental		Resistance Power		Healing Power	
Grade 1	⬭	Grade 1	⬭	Grade 1	⬭	Grade 1	⬭
Grade 2	⬭	Grade 2	⬭	Grade 2	⬭	Grade 2	⬭
Grade 3	⬭	Grade 3	⬭	Grade 3	⬭	Grade 3	⬭

E. Psychological Functions

E-1 **Speed**

	Quickly	Moderately	Slowly	Variably
Memorizing	⬭	⬭	⬭	⬭
Forgetfulness	⬭	⬭	⬭	⬭
Recalling	⬭	⬭	⬭	⬭
Initiation	⬭	⬭	⬭	⬭
Making new friends	⬭	⬭	⬭	⬭
Anger	⬭	⬭	⬭	⬭
Irritability	⬭	⬭	⬭	⬭

E-2 **Quality**

	Good / Firm/ Stable / High	Medium	Poor / Wavering / Unstable
Retaining	⬭	⬭	⬭
Planning	⬭	⬭	⬭
Execution	⬭	⬭	⬭
Achieving ends	⬭	⬭	⬭
Retaining friends	⬭	⬭	⬭
Anger	⬭	⬭	⬭
Forgiveness	⬭	⬭	⬭
Generosity	⬭	⬭	⬭
Faith and beliefs	⬭	⬭	⬭

E-3 **Memory type**

	Good	Poor
Olfactory	⬭	⬭
Auditory	⬭	⬭
Tactile	⬭	⬭
Gustatory	⬭	⬭
Visual	⬭	⬭
