# Supplementary material for: Whole genome expression and biochemical correlates of extreme constitutional types defined in Ayurveda
Source: J Transl Med. 2008 Sep 9;6:48. doi: 10.1186/1479-5876-6-48 (PMC2562368; doi:10.1186/1479-5876-6-48)
Supplement: Additional file 3 — Normal reference range for biochemical and hematological parameters. A table of standard accepted normal ranges in males and females for biochemical and hematological parameters. [file 1479-5876-6-48-S3.pdf]

Additional File 3. Normal reference range for biochemical and hematological parameters			
S.No	**TESTS**	Reference range (Female)	Reference range (Male)
1	Glucose ®	70-140 mg/dl	70-140 mg/dL
2	S.Uric acid	2.4-5.7 mg/dl	3.4-7.0 mg/dL
3	Prothrombin Time	11.5-15.5 sec.	11.5-15.5 sec.
4	Phosphorus	2.7-4.5 mg/dl	2.7-4.50 mg/dL
5	Copper	80-155 ug/dl	70-140 ug/dL
6	Zinc(ATOMIC ABSORP)	670-1240 ug/L	670-1240 ug/L
7	S.Ferritin	10-291ng/mL	22-322ng/mL
8	Haemoglobin	11.5-15.3 g/dl	14-17.4 g/dL
9	PCV	36-45 %	41.50-50.40 %
10	RBC count	4.5-5.1 mill/mm3	4.5-5.9 mill/mm3
11	Platelet count	150-450 thou/mm3	150-450 thou/mm3
12	Total Leucocytes	4.4-11.3 thou/mm3	4.4-11.3 thou/mm3
13	Neutrophils	45.5-74%	45.5-74.00 %
14	Lymphocytes	22.3-50 %	22.3-50 %
15	Monocytes	0.7-10 %	0. 7-10 %
16	Eosinophils	1-6%	1-6%
17	Basophils	<2%	<2%
18	ESR	0-20 mm/ 1 hr	0-15 mm/1 hr
19	S.prolactin	2.8-29.2 ng/ml (nonpreg.)	2.1-17.7 ng/mL
20	Total Cholesterol	140-200 mg/dl	133-200 mg/dL
21	TG	<150 mg/dl	<150 mg/dL
22	VLDL		<40
23	LDL	< 100 mg/dl	<130 mg/dL
24	HDL	45-70 mg/dl	35-60 mg/dL
25	Homocysteine	5-15 uM	5-15 uM
26	Vitamin B12	150-970 pg/ml	150-970 pg/ml
27	SGOT (AST)	<31 U/L	<37 U/L
28	SGPT (ALT)	<31 U/L	<41 U/L
29	Alkaline Phosphate	95-240 U/L	95-270 U/L
30	Serum Iron	37-145 ug/dl	59-158 ug/dL
31	Total Iron Binding Capacity	228-428 ug/dl	228-428 ug/dL
32	Transfarrin Saturation	16-45 %	16-45 %
33	GGPT	7-32 U/L	11-49 U/L
