# Supplementary material for: Whole genome expression and biochemical correlates of extreme constitutional types defined in Ayurveda
Source: J Transl Med. 2008 Sep 9;6:48. doi: 10.1186/1479-5876-6-48 (PMC2562368; doi:10.1186/1479-5876-6-48)
Supplement: Additional file 4 — Neighbor Joining tree showing relatedness of study population to the Indo European population. Heterogeneity and inter-relatedness of Indian populations among themselves and with study population (VPK) depicted in neighbour-joining tree illustrating population affinities based on Nei's DA distance. The study population are genetically closer to the Indo-European large populations predominantly from North India. The symbols represents linguistic Lineage (AA, Austro-Asiatic; IE, Indo-European; DR, Dravidian and TB, Tibeto-Burman) followed by geographical location (N, north; NE, north-east; W, west; E, east; S, south and C, central) and Ethnic category (LP, castes/large populations; SP, religious groups/small populations and IP, tribes/isolated population). [file 1479-5876-6-48-S4.pdf]

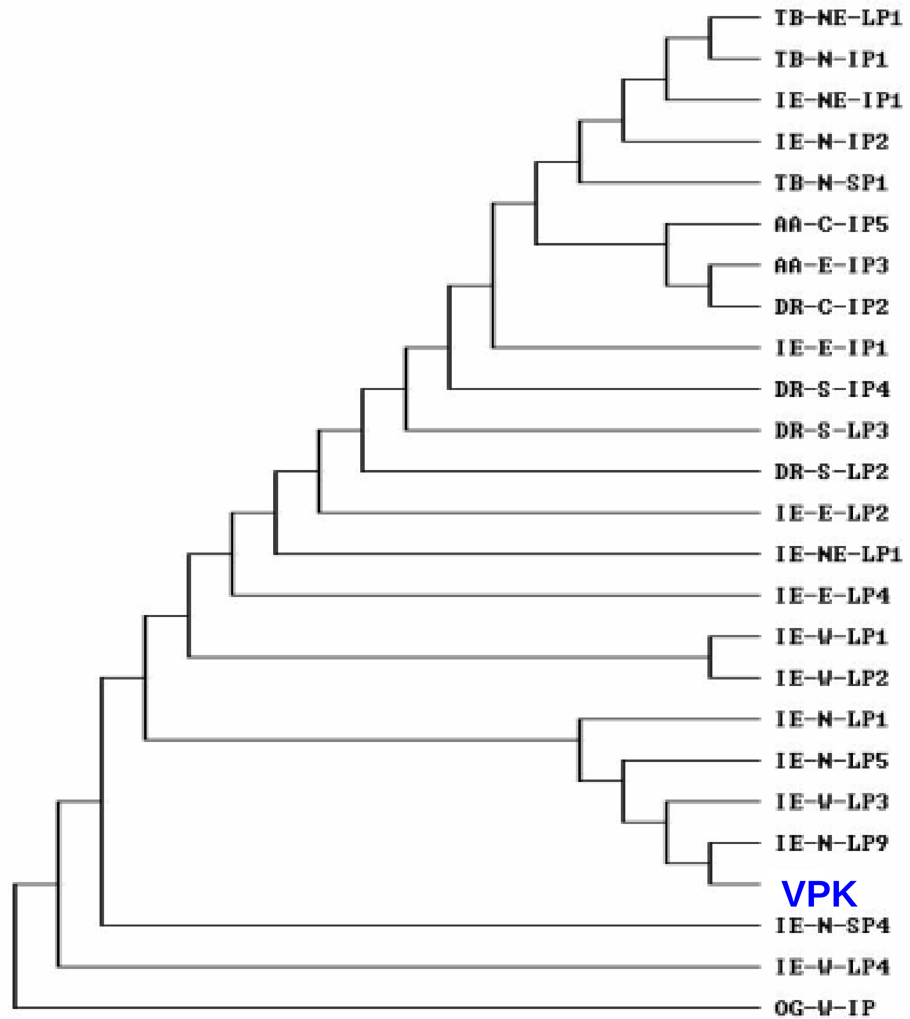

Additional File 4. Neighbor Joining tree showing relatedness of study population to the Indo European population
