# Supplementary material for: Whole genome expression and biochemical correlates of extreme constitutional types defined in Ayurveda
Source: J Transl Med. 2008 Sep 9;6:48. doi: 10.1186/1479-5876-6-48 (PMC2562368; doi:10.1186/1479-5876-6-48)
Supplement: Additional file 6 — Validation of biochemical differences amongst three Prakriti groups through analysis of significance performed in 1000 random iterations of same parent dataset in males. The boxplot of F-values of 1000 randomized comparisons of (A)Total cholesterol, (B) Triglycerides, (C) VLDL, (D) LDL, (E) LDL/HDL, (F) GGPT, (G) Serum Uric acid,(H) Prothrombin Time, (I) Hemoglobin, (J) PCV, (K) RBC, (L) Serum Zinc values. Thick bar indicated median F-value. Red spot is the mean F-value of randomized comparisons and green spot is the comparative mean F-value of V, P, K comparisons. [file 1479-5876-6-48-S6.pdf]

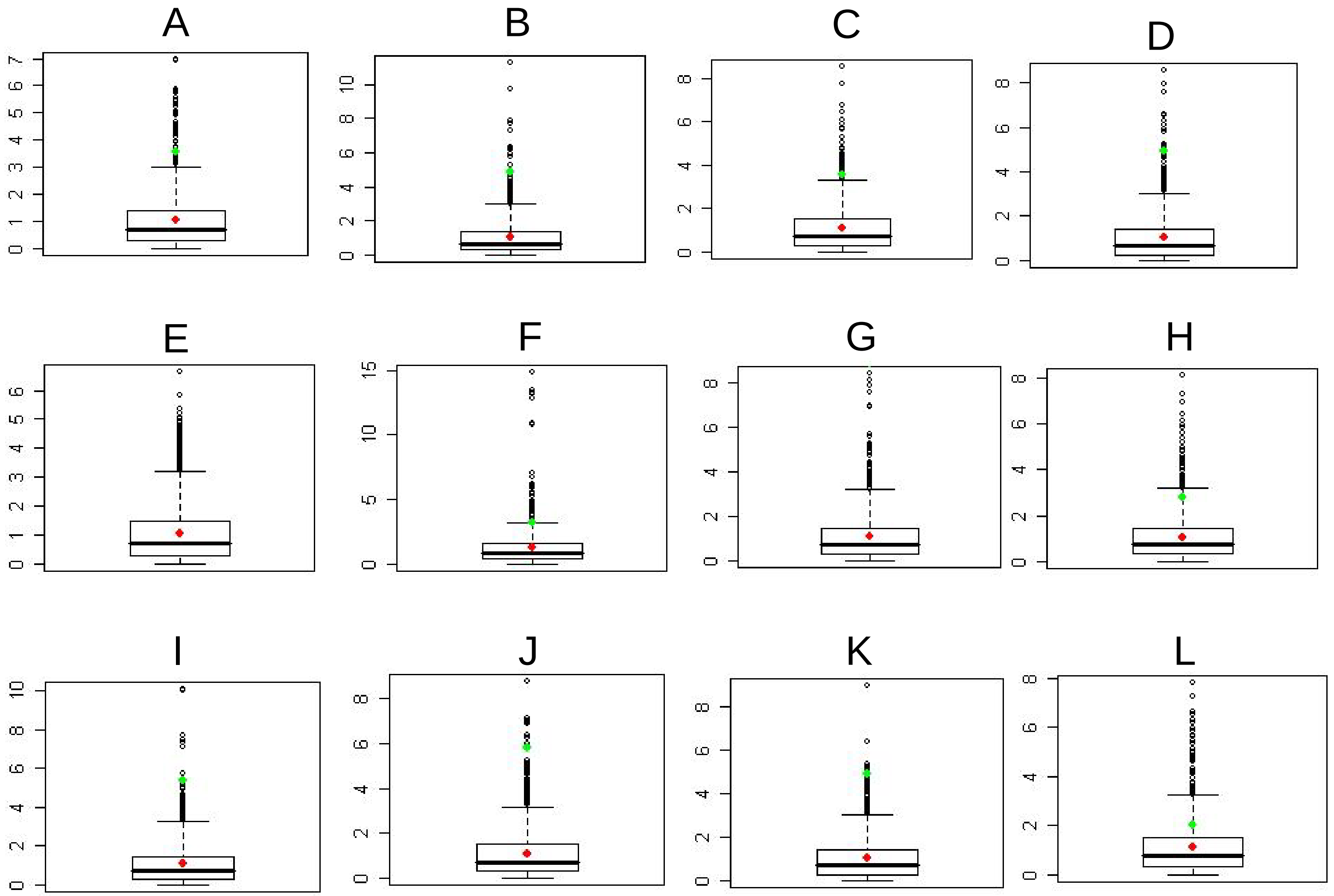

Additional File 6. Validation of biochemical differences amongst three Prakriti groups through analysis of significance performed in 1000 random iterations of same parent dataset in males The boxplot of F-values of 1000 randomized comparisons of (A)Total cholesterol, (B) Triglycerides, (C) VLDL, (D) LDL, (E) LDL/HDL, (F) GGPT, (G) Serum Uric acid,(H) Prothrombin Time, (I) Hemoglobin, (J) PCV, (K) RBC, (L) Serum Zinc values. Thick bar indicated median F-value. Red spot is the mean F-value of randomized comparisons and green spot is the comparative mean F-value of V, P, K comparisons
