# Supplementary material for: Whole genome expression and biochemical correlates of extreme constitutional types defined in Ayurveda
Source: J Transl Med. 2008 Sep 9;6:48. doi: 10.1186/1479-5876-6-48 (PMC2562368; doi:10.1186/1479-5876-6-48)
Supplement: Additional file 7 — List of genes differentially expressed among Prakriti types. The data represents a list of differentially expressed genes in males and females with information on their disease association as obtained from OMIM and GAD and their categorization as HUB and/Or Housekeeping genes. [file 1479-5876-6-48-S7.pdf]

Additional File 7:List of genes differentially expressed among Prakriti types

S.No	Gender	Profile	Acc	Symbol	Name	HUB genes	Housekeeping	Disease_OMIM	Disease_ GAD
1	Female	**V-K+P+**	T94105	ABCC1	ATP-binding cassette, sub-family C (CFTR/MRP), member 1			Earwax wet/dry	Cancer,Cardiovascular,Immune,Metabolic,Psychiatric
2	Female	V-K-P+	BG750060	AGBL5	ATP/GTP binding protein-like 5				
3	Female	V+K-P-	H64609	AHR	Aryl hydrocarbon receptor	HUB			Cancer,Developmental,Metabolic,Psychiatric,ReProduction,Vision
4	Female	V+K+P-	AA020896	ANKRD53	Ankyrin repeat domain 53				
5	Female	V+K-P+	N94268	ANPEP	Alanyl (membrane) aminopeptidase (aminopeptidase N, aminopeptidase M, microsomal aminopeptidase, CD13, p150)				Immune,Normal Variation
6	Female	V+K-P-	BG387747	API5	Apoptosis inhibitor 5		Housekeeping		
7	Female	V+K-P+	H95186	ARL16	ADP-ribosylation factor-like 16				
8	Female	V-K+P+	CX166694	ARL6IP6	ADP-ribosylation-like factor 6 interacting protein 6				
9	Female	V-K+P-	BM918995	ATP5G2	ATP synthase, H+ transporting, mitochondrial F0 complex, subunit C2 (subunit 9)				
10	Female	V+K-P+	AA053325	ATP6V1G1	ATPase, H+ transporting, lysosomal 13kDa, V1 subunit G1		Housekeeping		
11	Female	V+K-P+	T97408	BAG1	BCL2-associated athanogene	HUB			Neurological
12	Female	V+K-P+	N93740	BBS2	Bardet-Biedl syndrome 2			Bardet-Biedl syndrome 2	
13	Female	V+K+P-	BM992552	BIVM	Basic, immunoglobulin-like variable motif containing				
14	Female	V+K-P-	R89879	C10orf65	Chromosome 10 open reading frame 65				
15	Female	V-K+P+	R69813	C11orf49	Chromosome 11 open reading frame 49				
16	Female	V+K-P+	N99693	C12orf32	Chromosome 12 open reading frame 32				
17	Female	V+K-P+	N93440	C1orf38	Chromosome 1 open reading frame 38				
18	Female	V-K+P+	W02372	C20orf175	Chromosome 20 open reading frame 175				
19	Female	V-K-P+	T48696	C5orf32	Chromosome 5 open reading frame 32				
20	Female	V+K+P-	BM920182	C9orf16	Chromosome 9 open reading frame 16		Housekeeping		
21	Female	V+K-P+	H07996	C9orf91	Chromosome 9 open reading frame 91				

S.No	Gender	Profile	Acc	Symbol	Name	HUB genes	Housekeeping	Disease_OMIM	Disease_ GAD
22	Female	V+K+P-	AL120360	CAMK2B	Calcium/calmodulin-dependent protein kinase (CaM kinase) II beta	HUB			
23	Female	V-K+P-	BM924711	CD14	CD14 molecule				
24	Female	V-K+P-	N24337	CD44	CD44 molecule (Indian blood group)	HUB		Blood group	Immune
25	Female	V-K+P-	N24337	CD44	CD44 molecule (Indian blood group)	HUB		Blood group	Immune
26	Female	V+K-P+	N91433	CFH	Complement factor H			Factor H and factor H-like 1 (3),Hemolytic-uremic syndrome,Nephropathy,Macular degeneration age-related 1	Cardiovascular,Immune,Metabolic,Vision
27	Female	V-K+P-	N45640	CH25H	Cholesterol 25-hydroxylase				
28	Female	V-K-P+	BQ063705	CHCHD2	Coiled-coil-helix-coiled-coil-helix domain containing 2				
29	Female	V+K-P-	R00682	CLEC2D	C-type lectin domain family 2, member D				
30	Female	V-K+P-	N53883	DCUN1D4	DCN1, defective in cullin neddylation 1, domain containing 4 (S. cerevisiae)				
31	Female	V+K-P-	AA393337	KFZP564O05	Hypothetical protein DKFZp564O0523	HUB			
32	Female	V+K-P-	AA393337	KFZP564O05	Hypothetical protein DKFZp564O0523	HUB			
33	Female	V+K-P-	BM821533	KFZp686L141	Hypothetical gene supported by BX538329				
34	Female	V-K+P+	H89551	DSG2	Desmoglein 2				
35	Female	V+K+P-	R14533	FLJ35220	Hypothetical protein FLJ35220				
36	Female	V+K-P-	N92106	FYB	FYN binding protein (FYB-120/130)	HUB			
37	Female	V+K-P-	AA043597	GALNACT-2	Chondroitin sulfate GalNAcT-2				
38	Female	V+K-P+	H60117	HEL308	DNA helicase HEL308				
39	Female	V+K+P-	AA011481	HIST2H2BE	Histone cluster 2, H2be	HUB			
40	Female	V+K-P-	H62639	HSPA8	Heat shock 70kDa protein 8	HUB	Housekeeping		Cancer,Renal
41	Female	V-K+P-	R09292	IFIT1L	Interferon-induced protein with tetratricopeptide repeats 1-like				
42	Female	V+K-P-	N63988	IFIT2	Interferon-induced protein with tetratricopeptide repeats 2				
43	Female	V-K+P+	T77339	IL15RA	Interleukin 15 receptor, alpha				

S.No	Gender	Profile	Acc	Symbol	Name	HUB genes	Housekeeping	Disease_OMIM	Disease_ GAD
44	Female	V-K-P+	AW051744	IL1R2	Interleukin 1 receptor, type II				Immune,Infection,Neurological
45	Female	V+K+P-	W88639	ILK	Integrin-linked kinase	HUB	Housekeeping		
46	Female	V+K-P+	H03917	INSR	Insulin receptor	HUB		Diabetes mellitus insulin-resistant,Leprechaunism,Rabson-Mendenhall syndrome	Aging,Cardiovascular,Immune,Metabolic**
47	Female	V+K-P-	H18190	JAK1	Janus kinase 1 (a protein tyrosine kinase)	HUB	Housekeeping		Infection
48	Female	V-K-P+	T89217	JAM2	Junctional adhesion molecule 2				
49	Female	V-K+P-	BQ423875	KRAS	V-Ki-ras2 Kirsten rat sarcoma viral oncogene homolog	HUB		Bladder Cancer,Breast Cancer somatic,Leukemia,Lung Cancer,Noonan syndrome 3,Pancreatic carcinoma somatic,Stomach Cancer	Cancer*
50	Female	V+K+P+	BI116740	LOC126075	Hypothetical protein LOC126075				
51	Female	V+K+P-	H26642	LOC440498	Hypothetical gene supported by AK001829				
52	Female	V-K+P-	BF194709	LOC646324	Hypothetical LOC646324				
53	Female	V+K+P+	AA044796	LOC729096	Similar to BMS1-like, ribosome assembly protein				
54	Female	V+K+P-	AA040149	LOC730092	RRN3 RNA polymerase I transcription factor homolog (S. cerevisiae) pseudogene				
55	Female	V+K-P-	N20577	LRRC57	Leucine rich repeat containing 57				
56	Female	V+K+P+	AI096627	LRRC8A	Leucine rich repeat containing 8 family, member A				
57	Female	V-K+P-	N72242	LRRFIP1	Leucine rich repeat (in FLII) interacting protein 1				
58	Female	V-K-P+	W81346	MGAT1	Mannosyl (alpha-1,3-)-glycoprotein beta-1,2-N-acetylglucosaminyltransferase		Housekeeping		
59	Female	V+K-P+	R69953	MRPL19	Mitochondrial ribosomal protein L19				

S.No	Gender	Profile	Acc	Symbol	Name	HUB genes	Housekeeping	Disease_OMIM	Disease_ GAD
60	Female	V-K+P-	N57349	MRPL19	Mitochondrial ribosomal protein L19				
61	Female	V+K+P-	H10110	MRVI1	Murine retrovirus integration site 1 homolog				
62	Female	V-K-P+	AA043650	MYD88	Myeloid differentiation primary response gene (88)	HUB			
63	Female	V-K+P+	N31362	NF1	Neurofibromin 1 (neurofibromatosis, von Recklinghausen disease, Watson disease)			Hepatic adenoma,Leukemia acute promyelocytic,Leukemia juvenile myelomonocytic,MODY type III,Melanoma,Neurofibromatosis familial spinal,Neurofibromatosis,Neurofibromatosis-Noonan syndrome,Neutropenia severe congenital,Sertoli-cell-only syndrome,Stem-cell leukemia/lymphoma syndrome (3),Watson syndrome,Diabetes mellitus insulin-dependent,Diabetes mellitus noninsulin-dependent 2	Cancer,Cardiovascular,Psychiatric
64	Female	V-K+P-	W68069	NGFR	Nerve growth factor receptor (TNFR superfamily, member 16)	HUB			Psychiatric
65	Female	V+K-P-	AA149225	OSTF1	Osteoclast stimulating factor 1				
66	Female	V-K+P-	AA128349	PCBP1	Poly(rC) binding protein 1	HUB			
67	Female	V+K-P+	N72922	PDLIM5	PDZ and LIM domain 5				Psychiatric
68	Female	V+K-P+	AA233704	PIGT	Phosphatidylinositol glycan anchor biosynthesis, class T				

S.No	Gender	Profile	Acc	Symbol	Name	HUB genes	Housekeeping	Disease_OMIM	Disease_ GAD
69	Female	V-K+P-	BM477140	PLEK	Pleckstrin			Spinocerebellar ataxia 4 pure Japanese type,Age-related maculopathy susceptibility to	
70	Female	V-K+P+	H50917	PRIMA1	Proline rich membrane anchor 1				
71	Female	V-K+P+	AA033953	PRLR	Prolactin receptor	HUB			Cancer,Immune
72	Female	V+K-P+	R36315	PTPN12	Protein tyrosine phosphatase, non-receptor type 12	HUB		Colon Cancer (3)	
73	Female	V+K-P-	N72527	RAD51	RAD51 homolog (RecA homolog, E. coli) (S. cerevisiae)	HUB		Breast Cancer susceptibility to	Cancer*
74	Female	V+K-P-	AA040825	RANBP5	RAN binding protein 5	HUB			
75	Female	V+K+P-	R10548	RHD	Rh blood group, D antigen			Rh-negative blood type] (3)	Cardiovascular,Hematological,Psychiatric
76	Female	V+K+P-	BM009874	RPL21	Ribosomal protein L21				
77	Female	V-K+P-	BQ064037	RPS3A	Ribosomal protein S3A	HUB			
78	Female	V+K-P+	H38309	RUTBC2	RUN and TBC1 domain containing 2				
79	Female	V+K-P-	AA055241	S100A3	S100 calcium binding protein A3				
80	Female	V+K+P-	AA131244	SAC	Testicular soluble adenylyl cyclase			Epiphyseal dysplasia multiple 1,Pseudoachondroplasia,Spastic ataxia Charlevoix-Saguenay type,Hypercalciuria absorptive susceptibility to	
81	Female	V+K-P+	H03112	SAT1	Spermidine/spermine N1-acetyltransferase 1	HUB			
82	Female	V+K+P-	R83139	SCFV	Single-chain Fv fragment				
83	Female	V+K-P+	N99362	SCML2	Sex comb on midleg-like 2 (Drosophila)				
84	Female	V-K-P+	N29108	SEC63	SEC63 homolog (S. cerevisiae)			Polycystic liver disease	

S.No	Gender	Profile	Acc	Symbol	Name	HUB genes	Housekeeping	Disease_OMIM	Disease_ GAD
85	Female	V+K-P+	AA156247	SERPINB9	Serpin peptidase inhibitor, clade B (ovalbumin), member 9	HUB			
86	Female	V-K+P-	N48735	SHROOM4	Shroom family member 4				
87	Female	V+K-P+	H55836	SLC38A4	Solute carrier family 38, member 4				
88	Female	V+K-P+	N45602	STK4	Serine/threonine kinase 4				
89	Female	V+K+P-	AA115024	THRAP6	Thyroid hormone receptor associated protein 6				
90	Female	V-K+P-	BM970107	TXN	Thioredoxin	HUB	Housekeeping	Machado-Joseph disease,Spinocerebellar ataxia-1,Spinocerebellar ataxia-10 (3),Spinocerebellar ataxia-2,Spinocerebellar ataxia-7	
91	Female	V-K+P+	H89526	UGT8	UDP glycosyltransferase 8 (UDP-galactose ceramide galactosyltransferase)				
92	Female	V-K+P-	N56656	USPL1	Ubiquitin specific peptidase like 1				
93	Female	V+K-P-	AW959692	VEGFC	Vascular endothelial growth factor C				
94	Female	V+K-P-	AA203710	XPO7	Exportin 7		Housekeeping		
95	Female	V+K-P+	T66930	ZNF552	Zinc finger protein 552				
96	Female	V-K-P+	H84096	unannotated					
97	Female	V+K-P+	BQ025516	unannotated					
98	Female	V+K-P+	H20874	unannotated					
99	Female	V-K+P-	R18453	unannotated					
100	Female	V+K-P+	T67658	unannotated					
101	Female	V+K-P+	N94272	unannotated					
102	Female	V+K-P+	R96161	unannotated					
103	Female	V+K-P+	H29534	unannotated					
104	Female	V-K+P-	H68035	unannotated					
105	Female	V+K+P-	AA053901	unannotated					
106	Female	V+K-P-	W02639	unannotated					
107	Female	V+K-P-	H29340	unannotated					
108	Female	V+K-P-	H30223	unannotated					
109	Female	V-K+P+	H63707	unannotated					
110	Female	V-K+P-	H51552	unannotated					

S.No	Gender	Profile	Acc	Symbol	Name	HUB genes	Housekeeping	Disease_OMIM	Disease_ GAD
111	Female	V+K-P+	T82014	unannotated					
112	Female	V-K-P+	H49130	unannotated					
113	Female	V+K-P+	N80448	unannotated					
114	Female	V+K-P+	H58017	unannotated					
115	Female	V+K-P+	H29067	unannotated					
116	Female	V+K-P-	CR745150	unannotated					
117	Female	V+K-P+	W01982	unannotated					
118	Female	V+K-P+	W87872	unannotated					
119	Female	V+K-P+	H79213	unannotated					
120	Female	V+K-P+	N99795	unannotated					
121	Female	V+K-P+	R33062	unannotated					
122	Female	V+K-P-	T85040	unannotated					
123	Female	V-K-P+	W31733	unannotated					
124	Female	V+K-P-	R50526	unannotated					
125	Female	V+K-P+	W01596	unannotated					
126	Female	V+K-P+	BX102165	unannotated					
127	Female	V+K-P+	BF982646	unannotated					
128	Female	V+K-P+	N34765	unannotated					
129	Female	V+K-P+	N99429	unannotated					
130	Female	V+K-P+	R26325	unannotated					
131	Female	V+K-P+	N73624	unannotated					
132	Female	V+K-P+	AI357692	unannotated					
133	Female	V+K-P+	H53253	unannotated					
134	Female	V+K+P-	R11292	unannotated					
135	Female	V+K-P+	H87074	unannotated					
136	Female	V+K-P+	N57713	unannotated					
137	Female	V+K-P-	CR739860	unannotated					
138	Female	V+K-P+	T84199	unannotated					
139	Female	V+K+P-	N92095	unannotated					
140	Female	V+K-P+	R36342	unannotated					
141	Female	V+K-P+	R87818	unannotated					
142	Female	V-K+P-	AA203127	unannotated					
143	Female	V+K+P-	H91361	unannotated					
144	Female	V+K-P+	AW959279	unannotated					
145	Female	V+K-P+	AA005396	unannotated					
146	Female	V+K-P-	BX500906	unannotated					
147	Female	V+K-P-	H47541	unannotated					
148	Female	V+K-P+	T78481	unannotated					
149	Female	V-K+P-	H91306	unannotated					
150	Female	V+K-P+	H80655	unannotated					

S.No	Gender	Profile	Acc	Symbol	Name	HUB genes	Housekeeping	Disease_OMIM	Disease_ GAD
151	Female	V+K+P-	W31740	unannotated					
152	Female	V-K+P-	H47701	unannotated					
153	Female	V+K-P-	N50668	unannotated					
154	Female	V+K-P+	R09418	unannotated					
155	Female	V-K-P+	R71560	unannotated					
156	Female	V+K-P-	AA031783	unannotated					
157	Female	V+K-P+	R32718	unannotated					
158	Female	V+K-P+	AA203154	unannotated					
159	Female	V-K-P+	T75253	unannotated					
160	Female	V+K-P+	BM990616	unannotated					
161	Female	V-K+P-	AA044889	unannotated					
162	Female	V+K-P+	BG678236	unannotated					
163	Female	V+K-P+	H72480	unannotated					
164	Female	V+K-P+	T84203	unannotated					
165	Female	V+K-P-	N38787	unannotated					
166	Female	V-K-P+	H49610	unannotated					
167	Female	V-K+P-	AA040696	unannotated					
168	Female	V-K-P+	H89206	unannotated					
169	Female	V+K-P+	AA131265	unannotated					
170	Female	V+K-P-	AA101823	unannotated					
171	Female	V+K-P+	H77714	unannotated					
172	Female	V+K+P-	R86045	unannotated					
173	Female	V+K-P-	N33616	unannotated					
174	Female	V+K-P+	H64449	unannotated					
175	Female	V-K+P+	H73186	unannotated					
176	Female	V-K-P+	H86214	unannotated					
177	Female	V+K-P+	H55914	unannotated					
178	Female	V+K-P+	BX114578	unannotated					
179	Female	V+K-P+	H77714	unannotated					
180	Female	V+K-P+	N80982	unannotated					
181	Female	V+K-P-	AA210886	unannotated					
182	Female	V-K+P+	AA213442	unannotated					
183	Female	V+K-P+	H60107	unannotated					
184	Female	V+K-P+	H78546	unannotated					
185	Male	V+K-P+	H48691	AASS	Aminoadipate-semialdehyde synthase			Hyperlysinemia, Saccharopinuria	
186	Male	V-K+P+	BE835258	ADAMTSL4	ADAMTS-like 4	HUB			
187	Male	V+K-P+	H58086	ADCY7	Adenylate cyclase 7				

S.No	Gender	Profile	Acc	Symbol	Name	HUB genes	Housekeeping	Disease_OMIM	Disease_ GAD
188	Male	V-K+P+	W25455	ADM	Adrenomedullin			Acyl-CoA dehydrogenase medium chain deficiency of, Cowchock syndrome (2), Glomerulocystic kidney disease with hyperuricemia and isosthenuria, Hyperuricemic nephropathy familial juvenile, Kallmann syndrome (3), Macular dystrophy autosomal dominant chromosome 6-linked,Medullary cystic kidney disease 2 (2), Medullary cystic kidney disease 2, Stargardt disease 3	Cardiovascular,Metabolic
189	Male	V+K-P-	R84726	ADORA1	Adenosine A1 receptor				Cardiovascular,Psychiatric
190	Male	V-K+P-	BG750060	AGBL5	ATP/GTP binding protein-like 5				
191	Male	V+K+P-	BM561545	AHCTF1	AT hook containing transcription factor 1				
192	Male	V+K-P+	AA036635	AKAP10	A kinase (PRKA) anchor protein 10			Longevity reduced	Cardiovascular
193	Male	V-K+P-	AV747676	AKT3	V-akt murine thymoma viral oncogene homolog 3 (protein kinase B, gamma)				

S.No	Gender	Profile	Acc	Symbol	Name	HUB genes	Housekeeping	Disease_OMIM	Disease_ GAD
194	Male	V+K-P-	N42072	ALOX5AP	Arachidonate 5-lipoxygenase-activating protein			Myocardial infarction, Stroke susceptibility to	Cardiovascular,Immune
195	Male	V-K+P+	W05331	AMZ2	Archaemetzincins-2				
196	Male	V-K+P-	BG546113	ARTS-1	Type 1 tumor necrosis factor receptor shedding aminopeptidase regulator				Cardiovascular
197	Male	V+K-P+	N35460	ASH1L	Ash1 (absent, small, or homeotic)-like (Drosophila)				
198	Male	V+K-P+	W58264	ATP6V0B	ATPase, H+ transporting, lysosomal 21kDa, V0 subunit b		Housekeeping		
199	Male	V-K+P-	H82521	ATP6V0B	ATPase, H+ transporting, lysosomal 21kDa, V0 subunit b		Housekeeping		
200	Male	V+K-P+	T85906	BRD4	Bromodomain containing 4				
201	Male	V+K-P-	AA039928	C10orf128	Chromosome 10 open reading frame 128				
202	Male	V+K-P+	W68477	C1RL	Complement component 1, r subcomponent-like				
203	Male	V-K+P+	W24877	C20orf77	Chromosome 20 open reading frame 77				
204	Male	V+K+P-	BM790397	C6orf125	Chromosome 6 open reading frame 125				
205	Male	V+K+P-	R66945	C9orf150	Chromosome 9 open reading frame 150				
206	Male	V+K-P-	BI550180	CALM2					
207	Male	V+K+P-	BM683235	CD24	CD24 molecule			Multiple sclerosis susceptibility to	Immune
208	Male	V-K+P-	T87012	CD79A	CD79a molecule, immunoglobulin-associated alpha	HUB			
209	Male	V-K+P-	AA036972	CDC40	Cell division cycle 40 homolog (S. cerevisiae)				
210	Male	V+K-P-	BM463991	CDK5R1	Cyclin-dependent kinase 5, regulatory subunit 1 (p35)	HUB			
211	Male	V+K-P-	BQ945970	CDKN1C	Cyclin-dependent kinase inhibitor 1C (p57, Kip2)			Beckwith-Wiedemann syndrome	Metabolic
212	Male	V+K+P-	R55530	CHI3L1	Chitinase 3-like 1 (cartilage glycoprotein-39)				

S.No	Gender	Profile	Acc	Symbol	Name	HUB genes	Housekeeping	Disease_OMIM	Disease_ GAD
213	Male	V-K+P-	BE675690	COX10	COX10 homolog, cytochrome c oxidase assembly protein, heme A: farnesyltransferase (yeast)			Encephalopathy progressive mitochondrial	
214	Male	V+K-P+	BM717592	CSH2	Chorionic somatomammotropin hormone 2				Metabolic
215	Male	V-K+P-	W95228	CTSG	Cathepsin G	HUB			Cardiovascular,Neurological
216	Male	V-K+P+	CD108783	DDX5	DEAD (Asp-Glu-Ala-Asp) box polypeptide 5	HUB			
217	Male	V-K+P+	N94809	DPYD	Dihydropyrimidine dehydrogenase			Thymine-uraciluria (3),Fluorouracil toxicity	Cancer,Developmental,Metabolic,Normal Variation,Pharmacogenomics
218	Male	V+K-P+	BG337015	DUSP5	Dual specificity phosphatase 5				
219	Male	V+K+P-	H08650	EGLN1	Egl nine homolog 1 (C. elegans)				
220	Male	V+K-P-	AA149511	EGLN1	Egl nine homolog 1 (C. elegans)				
221	Male	V-K+P+	N43860	EXOSC9	Exosome component 9	HUB			

S.No	Gender	Profile	Acc	Symbol	Name	HUB genes	Housekeeping	Disease_OMIM	Disease_ GAD
222	Male	V+K-P+	H71213	F2	Coagulation factor II (thrombin)	HUB		BCG and salmonella infection disseminated, Bamforth-Lazarus syndrome, Bare lymphocyte syndrome type I due to TAP2 deficiency, Calcinosis tumoral, Charcot Marie-Tooth disease type 4B2, Chondrosarcoma, Chronic granulomatous disease due to deficiency of NCF-2, Complex V mitochondrial respiratory chain deficiency of, Coronary artery disease autosomal dominant 1, Diabetes mellitus noninsulin-dependent, Dysprothrombinemia (3), Fibromatosis gingival 2, Fibrosis of extraocular	Cancer,Cardiovascular**,Hematological,Immune,Metabolic,Reoroduction*

S.No	Gender	Profile	Acc	Symbol	Name	HUB genes	Housekeeping	Disease_OMIM	Disease_ GAD
223	Male	V-K-P+	AA031420	FAS	Fas (TNF receptor superfamily, member 6)	HUB		Advanced sleep phase syndrome familial, AutoImmune lymphoproliferative syndrome type IA, Squamous cell carcinoma burn scar-related, AutoImmune lymphoproliferative syndrome}, Systemic lupus erythematosus susceptibility	Aging,Cancer,Hematological,Immune ***,Infection,Metabolic,Neurological,Reoroduction
224	Male	V+K-P-	W55993	FBN2	Fibrillin 2 (congenital contractural arachnodactyly)			Contractural arachnodactyly	Cardiovascular,Developmental
225	Male	V-K-P+	H71637	FCER1G	Fc fragment of IgE, high affinity I, receptor for; gamma polypeptide				Cardiovascular,Immune,Renal
226	Male	V-K-P+	W92270	FCGR2A	Fc fragment of IgG, low affinity IIa, receptor (CD32)		Housekeeping	Lupus nephritis	Cancer,Cardiovascular,Hematological,Immune***,Infection*,Neurological,Renal
227	Male	V-K+P+	T87708	FGL2	Fibrinogen-like 2				Immune
228	Male	V-K+P+	BM998060	FGL2	Fibrinogen-like 2				Immune
229	Male	V+K-P-	AA046539	FLJ20186	Hypothetical protein FLJ20186				
230	Male	V-K+P-	T90037	FLJ20273	RNA-binding protein				
231	Male	V-K+P-	N48346	FNIP1	Folliculin interacting protein 1				
232	Male	V-K+P+	AA149521	FOXN3	Forkhead box N3				
233	Male	V-K+P+	BM852433	FTL	Ferritin, light polypeptide	HUB		Basal ganglia disease adult-onset, Epilepsy, Hyperferritinemia-cataract syndrome	Neurological

S.No	Gender	Profile	Acc	Symbol	Name	HUB genes	Housekeeping	Disease_OMIM	Disease_ GAD
234	Male	V-K+P+	BQ130731	FTL	Ferritin, light polypeptide	HUB		Basal ganglia disease adult-onset, Epilepsy, Hyperferritinemia-cataract syndrome	Neurological
235	Male	V+K-P-	N44024	FZD7	Frizzled homolog 7 (Drosophila)				
236	Male	V+K-P-	BM999387	GADD45A	Growth arrest and DNA-damage-inducible, alpha	HUB			
237	Male	V-K+P+	W52609	GLRX	Glutaredoxin (thioltransferase)				
238	Male	V-K+P+	N46532	GPR177	G protein-coupled receptor 177				
239	Male	V+K+P-	BM725539	GRHPR	Glyoxylate reductase/hydroxypyruvate reductase			Hyperoxaluria primary type II	
240	Male	V-K-P+	BQ128382	HBA1	Hemoglobin, alpha 1			Erythremias, Heinz body anemias, Methemoglobinemias, Thalassemias	Hematological,Immune,Infection
241	Male	V-K-P+	BQ130241	HBA1	Hemoglobin, alpha 1			Erythremias, Heinz body anemias, Methemoglobinemias, Thalassemias	Hematological,Immune,Infection
242	Male	V+K-P+	BQ028891	HBB	Hemoglobin, beta			Erythremias, HPFH, Heinz body anemias, Methemoglobinemias, Sickle cell anemia (3), Thalassemia-beta dominant inclusion-body, Thalassemias	Hematological,Infection,Metabolic

S.No	Gender	Profile	Acc	Symbol	Name	HUB genes	Housekeeping	Disease_OMIM	Disease_ GAD
243	Male	V+K-P+	BQ028891	HBB	Hemoglobin, beta			Erythremias, HPFH, Heinz body anemias, Methemoglobinemias, Sickle cell anemia (3), Thalassemia-beta dominant inclusion-body, Thalassemias	Hematological,Infection,Metabolic
244	Male	V+K-P+	H12367	HBB	Hemoglobin, beta			Erythremias, HPFH, Heinz body anemias, Methemoglobinemias, Sickle cell anemia (3), Thalassemia-beta dominant inclusion-body, Thalassemias	Hematological,Infection,Metabolic
245	Male	V+K-P+	BM996267	HBB	Hemoglobin, beta			Erythremias, HPFH, Heinz body anemias, Methemoglobinemias, Sickle cell anemia (3), Thalassemia-beta dominant inclusion-body, Thalassemias	Hematological,Infection,Metabolic
246	Male	V+K-P-	R77671	hCG_1646491	Brother of ataxin-1				
247	Male	V+K-P+	BM842766	HIATL1	Hippocampus abundant transcript-like 1				
248	Male	V+K-P+	W38744	HIPK3	Homeodomain interacting protein kinase 3	HUB			
249	Male	V+K-P-	H27034	HLA-C	Major histocompatibility complex, class I, C	HUB			Cancer,Hematological,Immune**,Infection*,Reproduction
250	Male	V+K-P-	H44273	HLA-C	Major histocompatibility complex, class I, C	HUB			Cancer,Hematological,Immune**,Infection*,Reproduction

S.No	Gender	Profile	Acc	Symbol	Name	HUB genes	Housekeeping	Disease_OMIM	Disease_ GAD
251	Male	V-K-P+	H25099	HLA-DQA1	Major histocompatibility complex, class II, DQ alpha 1				Cancer*,Cardiovascular,Developmental,Immune****,Infection*,Metabolic*,Neurological,Normal Variation,Psychiatric,Renal,Reoroduction
252	Male	V+K-P+	AA029822	HLA-DQB1	Major histocompatibility complex, class II, DQ beta 1			Creutzfeldt-Jakob disease variant resistance to, Multiple sclerosis susceptibility to	Cancer**,Cardiovascular,Hematological,Immune*****,Infection***,Metabolic*,Neurological,Normal Variation,Psychiatric*,Renal,Reoroduction*,Vision
253	Male	V-K+P-	AA149393	HPRT1	Hypoxanthine phosphoribosyltransferase 1 (Lesch-Nyhan syndrome)			HPRT-related gout, Lesch-Nyhan syndrome	Cancer,Chemical Dependancy,Metabolic
254	Male	V-K+P-	H30789	HSPC148	Hypothetical protein HSPC148				
255	Male	V+K-P-	R73278	ICAM3	Intercellular adhesion molecule 3				
256	Male	V-K-P+	N63988	IFIT2	Interferon-induced protein with tetratricopeptide repeats 2				
257	Male	V+K-P+	H49853	IFITM1	Interferon induced transmembrane protein 1 (9-27)		Housekeeping		
258	Male	V-K+P+	T96831	IFNAR1	Interferon (alpha, beta and omega) receptor 1	HUB			Immune,Infection,Metabolic,Psychiatric
259	Male	V+K-P+	H84616	IGF1R	Insulin-like growth factor 1 receptor	HUB		Intrauterine and postnatal growth retardation (3)	Aging,Cardiovascular,Metabolic,Reorodnction
260	Male	V+K-P-	H27044	IGHA1	Immunoglobulin heavy constant alpha 1				
261	Male	V+K+P-	R79618	IGHA1	Immunoglobulin heavy constant alpha 1				
262	Male	V+K+P-	H45128	IGHA1	Immunoglobulin heavy constant alpha 1				
263	Male	V+K-P-	H52350	IGHG1	Immunoglobulin heavy constant gamma 1 (G1m marker)				Immune
264	Male	V+K-P-	H45793	IGHG1	Immunoglobulin heavy constant gamma 1 (G1m marker)				Immune
265	Male	V+K-P-	AI146644	IGHG1	Immunoglobulin heavy constant gamma 1 (G1m marker)				Immune
266	Male	V+K-P-	R86079	IGL@	Immunoglobulin lambda locus				
267	Male	V+K+P-	R49901	IGL@	Immunoglobulin lambda locus				

S.No	Gender	Profile	Acc	Symbol	Name	HUB genes	Housekeeping	Disease_OMIM	Disease_ GAD
268	Male	V+K-P-	R49901	IGL@	Immunoglobulin lambda locus				
269	Male	V+K-P-	H26788	IGL@	Immunoglobulin lambda locus				
270	Male	V-K-P+	W37552	IL11RA	Interleukin 11 receptor, alpha				Immune
271	Male	V+K-P+	R52482	IMPA2	Inositol(myo)-1(or 4)-monophosphatase 2				Psychiatric
272	Male	V-K+P-	AA115289	KALRN	Kalirin, RhoGEF kinase				
273	Male	V-K+P-	CA488240	KCNG1	Potassium voltage-gated channel, subfamily G, member 1				
274	Male	V+K-P+	W38679	KCNJ2	Potassium inwardly-rectifying channel, subfamily J, member 2	HUB		Long QT syndrome-7	
275	Male	V-K-P+	BM806879	KCNJ2	Potassium inwardly-rectifying channel, subfamily J, member 2	HUB		Long QT syndrome-7	
276	Male	V+K-P+	W38679	KCNJ2	Potassium inwardly-rectifying channel, subfamily J, member 2	HUB		Long QT syndrome-7	
277	Male	V-K+P+	AI951455	KIAA0040	KIAA0040				
278	Male	V-K+P-	R60601	KIAA0372	KIAA0372				

S.No	Gender	Profile	Acc	Symbol	Name	HUB genes	Housekeeping	Disease_OMIM	Disease_ GAD
279	Male	V+K-P+	AA024415	KRT1	Keratin 1 (epidermolytic hyperkeratosis)			Cirrhosis, Cyclic ichthyosis with epidermolytic hyperkeratosis, Epidermolysis bullosa simplex Koebner Dowling-Meara and Weber-Cockayne types, Epidermolysis bullosa simplex recessive, Epidermolytic hyperkeratosis, Epidermolytic hyperkeratosis, Ichthyosis histrix Curth-Macklin type, Ichthyosis cyclic with epidermolytic hyperkeratosis, Keratosis palmoplantaria striata, Keratosis palmoplantaris striata III, Meesmann corneal dystrophy, Nevus epidermal epidermolytic hyperkeratotic type, Pachyonychia congenita	
280	Male	V-K+P-	H95300	LCP1	Lymphocyte cytosolic protein 1 (L-plastin)				
281	Male	V-K+P-	AA203281	LDHD	Lactate dehydrogenase D				
282	Male	V+K+P-	R61021	LOC145899	Hypothetical protein LOC145899				
283	Male	V+K-P+	R16402	LOC197135	Hypothetical LOC197135				
284	Male	V+K-P+	H87076	LOC401152	HCV F-transactivated protein 1				
285	Male	V+K-P+	BQ021273	MEMO1	Mediator of cell motility 1				

S.No	Gender	Profile	Acc	Symbol	Name	HUB genes	Housekeeping	Disease_OMIM	Disease_ GAD
286	Male	V+K-P-	W81346	MGAT1			Housekeeping		
287	Male	V+K-P+	H47934	MGC12760	Hypothetical protein MGC12760				
288	Male	V-K+P-	AA063134	MGC27345	Hypothetical protein MGC27345				
289	Male	V-K+P+	AA213820	MGC52498	Hypothetical protein MGC52498				
290	Male	V-K-P+	N22335	MITD1	MIT, microtubule interacting and transport, domain containing 1			7974 Leukemia	
291	Male	V-K+P+	AI522083	MLL	Myeloid/lymphoid or mixed-lineage leukemia (trithorax homolog, Drosophila)	HUB			Cancer
292	Male	V-K+P+	R77519	MNDA	Myeloid cell nuclear differentiation antigen				
293	Male	V-K+P+	BM799719	MRPL35	Mitochondrial ribosomal protein L35				
294	Male	V+K-P+	R69774	MUC15	Mucin 15, cell surface associated				
295	Male	V+K-P-	AA099821	MYADM	Myeloid-associated differentiation marker				
296	Male	V+K-P-	H06796	MYLIP	Myosin regulatory light chain interacting protein				
297	Male	V+K-P+	H06796	MYLIP	Myosin regulatory light chain interacting protein				
298	Male	V+K+P-	T97240	MYO5C	Myosin VC				
299	Male	V+K-P+	R67302	NBEA	Neurobeachin				
300	Male	V+K+P-	AL527869	NCAPG2	Non-SMC condensin II complex, subunit G2				
301	Male	V+K-P-	R25036	NDST1	N-deacetylase/N-sulfotransferase (heparan glucosaminyl) 1				
302	Male	V+K-P-	BQ054257	NDUFA11	NADH dehydrogenase (ubiquinone) 1 alpha subcomplex, 11, 14.7kDa				
303	Male	V-K+P+	H70961	NFKBIZ	Nuclear factor of kappa light polypeptide gene enhancer in B-cells inhibitor, zeta				
304	Male	V-K+P+	N34884	NIF3L1	NIF3 NGG1 interacting factor 3-like 1 (S. pombe)	HUB			
305	Male	V-K-P+	H88805	NOV	Nephroblastoma overexpressed gene	HUB		Niemann-pick disease type C2	
306	Male	V+K-P+	BQ128229	NPC2	Niemann-Pick disease, type C2				
307	Male	V+K+P-	AL525867	NPL	N-acetylneuraminate pyruvate lyase (dihydrodipicolinate synthase)				

S.No	Gender	Profile	Acc	Symbol	Name	HUB genes	Housekeeping	Disease_OMIM	Disease_ GAD
308	Male	V+K-P+	AW138141	NRM	Nurim (nuclear envelope membrane protein)				
309	Male	V+K-P+	R69654	OSBPL3	Oxysterol binding protein-like 3				
310	Male	V+K-P+	R23871	PCBP1	Poly(rC) binding protein 1	HUB			
311	Male	V+K+P-	W32160	PCDHA6	Protocadherin alpha 6			Zellweger syndrome	
312	Male	V+K-P+	R76845	PEX14	Peroxisomal biogenesis factor 14				
313	Male	V+K-P+	W19988	PFAAP5	Phosphonoformate immuno-associated protein 5				
314	Male	V-K-P+	AA131389	PHACTR4	Phosphatase and actin regulator 4				
315	Male	V-K+P+	R02688	PHF21A	PHD finger protein 21A			Polycystic kidney disease adult	
316	Male	V+K-P+	W24775	PKD2	Polycystic kidney disease 2 (autosomal dominant)				Renal
317	Male	V+K-P-	BQ002836	PLXNB2	Plexin B2				
318	Male	V+K-P+	BG763627	PNPLA6	Patatin-like phospholipase domain containing 6				
319	Male	V+K-P+	W78000	POMZP3	POM (POM121 homolog, rat) and ZP3 fusion				
320	Male	V-K+P+	R80587	PPP2R2A	Protein phosphatase 2 (formerly 2A), regulatory subunit B, alpha isoform				
321	Male	V-K+P-	N92208	PPP4R2	Protein phosphatase 4, regulatory subunit 2				
322	Male	V+K+P-	AA018979	PRKACB	Protein kinase, cAMP-dependent, catalytic, beta				
323	Male	V+K+P-	AA128571	PRKCH	Protein kinase C, eta				
324	Male	V+K-P+	R99962	PTGS1	Prostaglandin-endoperoxide synthase 1 (prostaglandin G/H synthase and cyclooxygenase)				Cancer,Cardiovascular,Immune,Psychiatric
325	Male	V-K+P+	R80322	PTGS2	Prostaglandin-endoperoxide synthase 2 (prostaglandin G/H synthase and cyclooxygenase)				Cancer*,Cardiovascular,Hematological,Immune,Metabolic,Neurological,Psychiatric
326	Male	V-K+P-	AL575253	PVRL2	Poliovirus receptor-related 2 (herpesvirus entry mediator B)				Cardiovascular
327	Male	V+K+P-	N54827	RABGAP1L	RAB GTPase activating protein 1-like			Osteolysis familial expansile, Paget disease of bone	

S.No	Gender	Profile	Acc	Symbol	Name	HUB genes	Housekeeping	Disease_OMIM	Disease_ GAD
328	Male	V-K+P-	BQ070782	RAN	RAN, member RAS oncogene family	HUB	Housekeeping		
329	Male	V-K-P+	T84264	RAP2B	RAP2B, member of RAS oncogene family				
330	Male	V+K+P-	BE551027	RAPGEF2	Rap guanine nucleotide exchange factor (GEF) 2				
331	Male	V-K+P-	AI500475	RASGRP1	RAS guanyl releasing protein 1 (calcium and DAG-regulated)			Cone-rod dystrophy 3, Fundus flavimaculatus, Macular degeneration age-related 2, Retinitis pigmentosa-19, Stargardt disease-1, Schizophrenia	
332	Male	V+K-P-	H93740	RD3	Retinal degeneration 3				
333	Male	V-K+P-	R60831	RGMB	RGM domain family, member B				
334	Male	V+K+P-	N77152	RGS18	Regulator of G-protein signalling 18				
335	Male	V+K+P-	AA195206	RGS18	Regulator of G-protein signalling 18			Cavernous malformations of CNS and retina, Cerebral cavernous malformations-1, Hyperkeratotic cutaneous capillary-venous malformations associated with cerebral capillary malformations	
336	Male	V+K+P-	AA055094	RIT1	Ras-like without CAAX 1	HUB			
337	Male	V+K-P-	R13557	RPA2	Replication protein A2, 32kDa	HUB	Housekeeping		
338	Male	V+K-P+	R72766	RPS15	Ribosomal protein S15		Housekeeping		
339	Male	V-K+P-	AA534515	RPS3A	Ribosomal protein S3A	HUB			
340	Male	V+K-P-	BQ073203	S100A10	S100 calcium binding protein A10	HUB			

S.No	Gender	Profile	Acc	Symbol	Name	HUB genes	Housekeeping	Disease_OMIM	Disease_ GAD
341	Male	V-K+P-	R62721	SCYE1	Small inducible cytokine subfamily E, member 1 (endothelial monocyte-activating)				
342	Male	V+K+P-	W37168	SFRS2	Splicing factor, arginine/serine-rich 2	HUB	Housekeeping		
343	Male	V+K-P-	N49623	SMAP1L	Stromal membrane-associated protein 1-like				
344	Male	V+K+P-	H43425	SNX12	Sorting nexin 12				
345	Male	V+K-P-	BG942204	SON	SON DNA binding protein				
346	Male	V+K-P-	AA193339	SRRM1	Serine/arginine repetitive matrix 1		Housekeeping		
347	Male	V+K-P+	R88696	ST6GALNAC4	ST6 (alpha-N-acetyl-neuraminyl-2,3-beta-galactosyl-1,3)-N-acetylgalactosaminide alpha-2,6-sialyltransferase 4			hormone insensitivity with immunodeficiency, Leukemia acute promyeloyctic	
348	Male	V-K-P+	BI520110	STAT5B	Signal transducer and activator of transcription 5B	HUB			
349	Male	V-K+P+	N67807	STK16	Serine/threonine kinase 16				
350	Male	V+K-P-	R63027	STK40	Serine/threonine kinase 40				
351	Male	V-K+P+	R59251	SUV39H1	Suppressor of variegation 3-9 homolog 1 (Drosophila)	HUB			
352	Male	V+K-P+	AA156587	SYNC1	Syncoilin, intermediate filament 1			Transaldolase deficiency	
353	Male	V-K+P+	N24276	TALDO1	Transaldolase 1		Housekeeping		
354	Male	V+K-P+	R73296	TBC1D17	TBC1 domain family, member 17	HUB			
355	Male	V+K+P-	AA045267	TIE1	Tyrosine kinase with immunoglobulin-like and EGF-like domains 1			Endotoxin hyporesponsiveness (3)	
356	Male	V-K+P+	N42522	TLR4	Toll-like receptor 4	HUB			Cancer,Cardiovascular*,Immune**,Infection**,Metabolic,Neurological,Reproduction,Vision
357	Male	V-K+P+	N49034	TMEM66	Transmembrane protein 66				
358	Male	V-K-P+	R16934	TNFSF13B	Tumor necrosis factor (ligand) superfamily, member 13b				Immune
359	Male	V-K+P+	R16934	TNFSF13B	Tumor necrosis factor (ligand) superfamily, member 13b				Immune
360	Male	V-K+P-	R23917	TREX1	Three prime repair exonuclease 1				
361	Male	V-K+P+	R14194	TRIM66	Tripartite motif-containing 66				

S.No	Gender	Profile	Acc	Symbol	Name	HUB genes	Housekeeping	Disease_OMIM	Disease_ GAD
362	Male	V+K-P+	H08988	USP7	Ubiquitin specific peptidase 7 (herpes virus-associated)	HUB			
363	Male	V+K-P+	AW965088	ZKSCAN2	Zinc finger with KRAB and SCAN domains 2				
364	Male	V+K+P-	W58007	ZNF516	Zinc finger protein 516				
365	Male	V+K-P+	BX098130	unannotated					
366	Male	V+K-P+	R97949	unannotated					
367	Male	V-K+P-	AA213450	unannotated					
368	Male	V+K+P-	H39539	unannotated					
369	Male	V+K+P-	BM837817	unannotated					
370	Male	V+K+P-	N92095	unannotated					
371	Male	V-K+P-	AA021582	unannotated					
372	Male	V-K+P-	R26331	unannotated					
373	Male	V-K+P+	AA055986	unannotated					
374	Male	V-K+P-	R06606	unannotated					
375	Male	V+K+P-	R06500	unannotated					
376	Male	V+K-P+	H52273	unannotated					
377	Male	V-K+P-	AV711358	unannotated					
378	Male	V+K-P+	R34503	unannotated					
379	Male	V+K+P-	R55451	unannotated					
380	Male	V-K+P+	N63789	unannotated					
381	Male	V+K-P-	R20110	unannotated					
382	Male	V+K-P+	R20974	unannotated					
383	Male	V-K+P+	R83560	unannotated					
384	Male	V+K-P-	R55451	unannotated					
385	Male	V-K+P+	H86049	unannotated					
386	Male	V+K-P-	W78898	unannotated					
387	Male	V-K+P+	T93598	unannotated					
388	Male	V+K-P+	T83917	unannotated					
389	Male	V+K-P+	T86264	unannotated					
390	Male	V+K-P+	R84397	unannotated					
391	Male	V+K-P+	R09976	unannotated					
392	Male	V+K-P-	R87739	unannotated					
393	Male	V+K-P-	T85855	unannotated					
394	Male	V+K-P+	H02366	unannotated					
395	Male	V+K-P+	R59360	unannotated					
396	Male	V+K-P+	R97958	unannotated					
397	Male	V-K-P+	T80910	unannotated					
398	Male	V+K-P-	N92185	unannotated					
399	Male	V-K-P+	R91270	unannotated					

S.No	Gender	Profile	Acc	Symbol	Name	HUB genes	Housekeeping	Disease_OMIM	Disease_ GAD
400	Male	V+K-P+	T96870	unannotated					
401	Male	V-K+P+	H12953	unannotated					
402	Male	V+K+P-	H62088	unannotated					
403	Male	V+K-P+	T82117	unannotated					
404	Male	V+K-P+	T98701	unannotated					
405	Male	V+K-P+	AA147561	unannotated					
406	Male	V+K-P+	R79487	unannotated					
407	Male	V+K-P+	T91738	unannotated					
408	Male	V+K+P-	N43942	unannotated					
409	Male	V+K-P-	AA203276	unannotated					
410	Male	V+K-P+	H51271	unannotated					
411	Male	V+K-P+	T94951	unannotated					
412	Male	V+K-P+	H48738	unannotated					
413	Male	V+K+P-	N68486	unannotated					
414	Male	V+K-P+	R41373	unannotated					
415	Male	V+K-P+	N66410	unannotated					
416	Male	V+K-P-	T77494	unannotated					
417	Male	V-K-P+	H89240	unannotated					
418	Male	V+K-P+	T85771	unannotated					
419	Male	V-K+P-	R11282	unannotated					
420	Male	V+K-P+	R05895	unannotated					
421	Male	V+K-P+	AA099171	unannotated					
422	Male	V+K-P-	R19725	unannotated					
423	Male	V+K-P+	R13857	unannotated					
424	Male	V+K-P+	R78322	unannotated					
425	Male	V-K-P+	BQ049843	unannotated					
426	Male	V+K-P+	H51850	unannotated					
427	Male	V-K+P-	H61972	unannotated					
428	Male	V+K-P-	AA058635	unannotated					
