# Supplementary material for: Whole genome expression and biochemical correlates of extreme constitutional types defined in Ayurveda
Source: J Transl Med. 2008 Sep 9;6:48. doi: 10.1186/1479-5876-6-48 (PMC2562368; doi:10.1186/1479-5876-6-48)
Supplement: Additional file 8 — Real time validation of differentially expressed genes from microarray experiments. The data depicts the list of differentially expressed genes validated using quantitative real time PCR with 18S rRNA as internal control. Analysis has been carried out in 96 individual samples and P ≤ 0.05 has been considered significant. [file 1479-5876-6-48-S8.pdf]

	**Additional File 8. Validation of differentially expressed genes from microarray experiments**						
				**Test of significance between profiles in RT experiments**			
**S.No**	**Validated Genes**	**Microarray profiles in males**	**Microarray profiles in female**	**VK**	**KP**	**PV**	**VPK**
1	ADM	V-K+P+		0.01	NS	0.01	0.01
2	ATP5G2	V+K-P-	V-K+P-	0.04	NS	0.06	NS
3	CH25H	V-K+P+	V-K+P-	0.02	NS	NS	NS
4	FAS	V-K-P+		0.05	NS	0.05	NS
5	FTL	V-K+P+		0.02	NS	0.04	0.05
6	HLA-DQB1	V+K-P+		0.05	NS	0.01	0.02
7	KCNJ2	V+K-P+		NS	NS	0.05	NS
8	TALDO1	V-K+P+		NS	NS	0.04	NS
	* Analysis has been combined for male and female samples						
