# Supplementary material for: Whole genome expression and biochemical correlates of extreme constitutional types defined in Ayurveda
Source: J Transl Med. 2008 Sep 9;6:48. doi: 10.1186/1479-5876-6-48 (PMC2562368; doi:10.1186/1479-5876-6-48)
Supplement: Additional file 10 — Disease gene network of differentially expressed genes among Prakriti groups. The figure represents networks of differentially expressed genes and their disease associations obtained from Genetic Association Database (GAD) and OMIM in males (A) and females (B). Networks have been depicted using Cytoscape version 2.4.1 . The nodes colored in yellow represent disease category and those in pink depict gene symbols. Hub genes are represented by pink square nodes. The most connected genes are placed in the centre of the network. [file 1479-5876-6-48-S10.pdf]

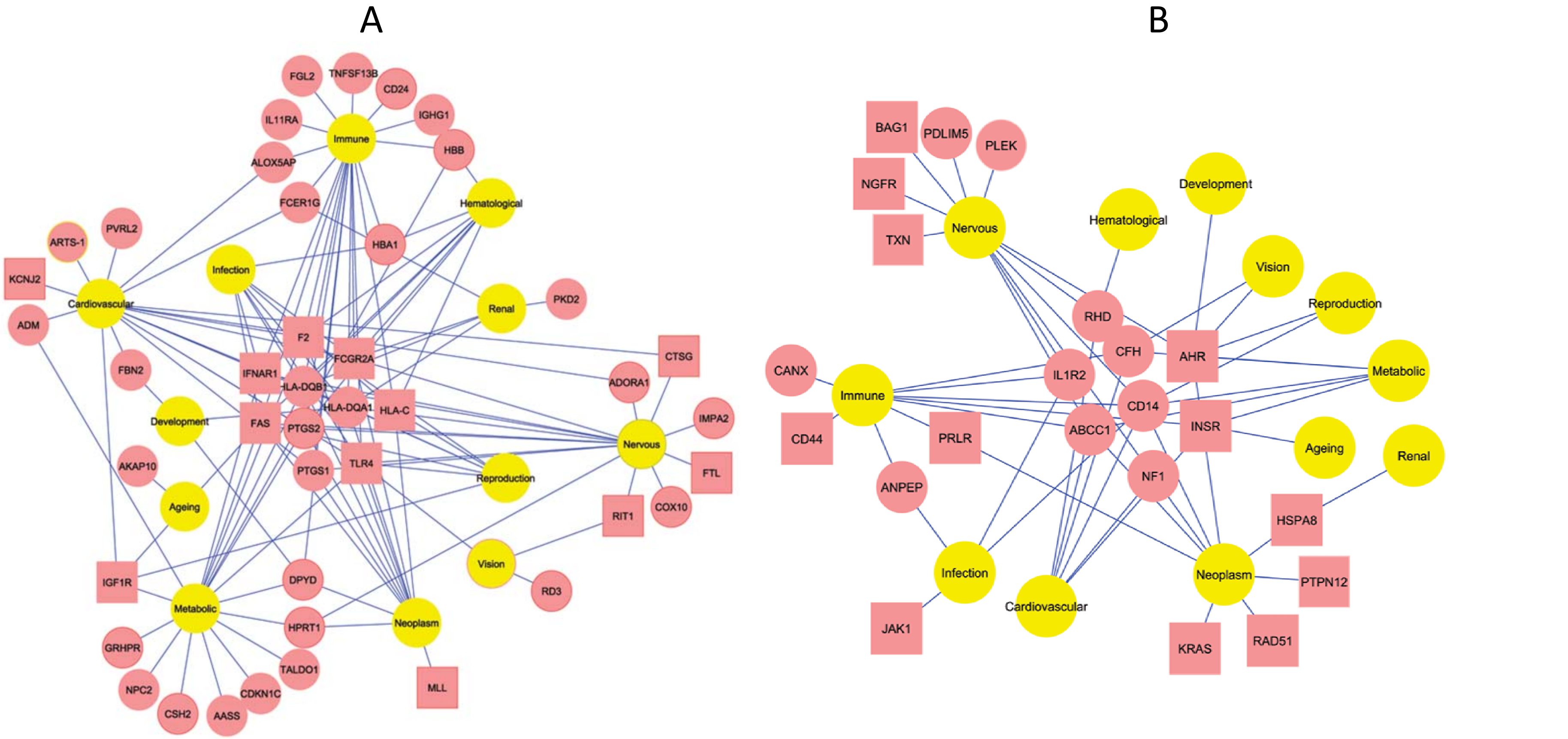

Additional File 10. Disease gene network of differentially expressed genes among *Prakriti* groups Networks of differentially expressed genes and their disease associations obtained from Genetic Association Database (GAD) and OMIM in males (A) and females (B). Networks have been depicted using Cytoscape version 2.4.1 (**http://www.cytoscape.org**). The nodes colored in yellow represent disease category and those in pink depict gene symbols. Hub genes are represented by pink square nodes. The most connected genes are placed in the centre of the network.
